# Clusters of hairpins induce intrinsic transcription termination in bacteria

**DOI:** 10.1038/s41598-021-95435-3

**Published:** 2021-08-10

**Authors:** Swati Gupta, Debnath Pal

**Affiliations:** Department of Computational and Data Sciences, Indian Institute of Science, Bangalore, Karnataka 560012 India

**Keywords:** Computational biology and bioinformatics, Transcription

## Abstract

Intrinsic transcription termination (ITT) sites are currently identified by locating single and double-adjacent RNA hairpins downstream of the stop codon. ITTs for a limited number of genes/operons in only a few bacterial genomes are currently known. This lack of coverage is a lacuna in the existing ITT inference methods. We have studied the inter-operon regions of 13 genomes covering all major phyla in bacteria, for which good quality public RNA-seq data exist. We identify ITT sites in 87% of cases by predicting hairpin(s) and validate against 81% of cases for which the *RNA-seq derived* sites could be calculated. We identify 72% of these sites correctly, with 98% of them located ≤ 80 bases downstream of the stop codon. The predicted hairpins form a *cluster* (when present < 15 bases) in two-thirds of the cases, the remaining being *single* hairpins. The largest number of *clusters* is formed by two hairpins, and the occurrence decreases exponentially with an increasing number of hairpins in the *cluster*. Our study reveals that hairpins form an effective ITT unit when they act in concert in a *cluster*. Their pervasiveness along with *single* hairpin terminators corroborates a wider utilization of ITT mechanisms for transcription control across bacteria.

## Introduction

Transcription is the first step of decoding genomic information and is initiated post recruitment of the RNA polymerase (RNAP) at the DNA start site^1,2^. It is continued through the elongation and termination steps that are tightly regulated. Any obstruction of transcription could be a pause, but a pause followed by the dismantling of the transcription complex causes transcription termination^3^. The cause of termination could be intrinsic^4^ or extrinsic^5^. Although many facets of the intrinsic transcription termination (ITT) driven by product mRNA are understood^6^, the common determinants required for such mechanisms to operate across bacterial organisms remain unclear.

During ITT, RNAP translocation on DNA is stalled by the product mRNA synthesized due to hairpin(s) formed from GC rich inverted repeat sequence stabilized by more number of hydrogen bonds^7,8^. Poly U tract, located immediately after the end of a hairpin, drive detachment of the RNA-polymerase from mRNA-genome complex^9–11^. Strong mRNA hairpins can also allosterically alter the RNAP structure to cause ITT^12^. Several algorithms, such as by Carafa et al.^13^, Brendel et al.^14^, or software like TransTerm^15^, RNAMotif^16^, and RNIE^17^ try to capture these genomic features to detect termination-capable hairpins. However, bacterial genome sequence analysis has shown the intergenic continuous poly U tract (in nascent RNA) to be infrequently present downstream of the stop codon. The 26,000 best candidate hairpins observed immediately downstream of the stop codon across 1060 bacterial species did not have any U stretch in ~ 50% cases^18^.

By our analysis and others^19–21^, it was observed by computational as well as in vivo methods that poly U trail is not necessary for ITT. In *M. tuberculosis*, nearly 90% of such terminator structures are found either without a U trail or with a mixed U/A trail, unlike the trends in *E. coli* where many studies claim the importance of downstream U trail. Other organisms with such findings include *Streptomyces, H. pylori*^22–24^. Even some genes in *E. coli* show that termination efficiency only reduces slightly when the poly U trail is reduced^25,26^.

Other features in sequences downstream of the stop codon also affect termination efficiency along with stem-loop and poly U segment downstream of the hairpin stem. This includes two G/C base pairs in the U-rich tract, an elemental pause sequence, or conserved sequences like TCTG downstream of hairpin^27,28^. A summary of the pertinent literature is given in Supplementary Table S1.

The RNA hairpins thus far identified as transcription terminators have been classified in a bid to understand their secondary structure and function^18^. Classification labels such as I- and L-shape have been given to *single* hairpins where L shape hairpins are I hairpins extended by a poly-U trail. When consecutive I hairpins are separated by a short RNA sequence, they are classified as U-, or V-shaped when there is no separation, and X-shaped when coded by the opposite DNA strands. U-, V-shaped hairpins are less frequent with only 10% and 15% cases found in *M. tuberculosis* and *E. coli*, respectively^21^, although they are deemed equally effective as the I or L-shaped hairpins^29^. A survey on 1060 genomes found only about one-third of the genes to have a hairpin downstream of the stop codon, and of these, 86% I/L-shaped and the remaining U/X/V-shaped (9%/ 5%/0.1% occurrence, respectively) made up the whole composition^18^. How the remaining two-third of genes are accomplishing transcription termination is the key question to be answered. If these happen through extrinsic termination mechanisms, proteins like the Rho and NusA need to be commonly overexpressed or conditionally overexpressed relative to RNAP. The published literature does not document such facts, especially when the hexameric form of the Rho protein is known to be required for fruitful transcription termination (further details in “Discussion”).

In this paper, we attempt to understand how the remaining two-third of operons in microbial genomes are accomplishing their transcription termination. We propose a *cluster* of hairpins to fill this gap. The *cluster*s are defined as a set of short contiguous hairpins located within < 15 bases of each other and the others as *single*. By short hairpin, we refer to hairpins with a stem length of 2–10 base pairs. Hairpins with stem lengths larger than this are long hairpins. Each such *cluster* is hypothesized to stall the RNAP to drive a successful ITT. The presence of a poly U trail is not essential for successful termination. We validate our paradigm using 13 genomes from diverse bacterial phyla. We can correctly identify that around three-fourths of all microbial operons/single genes have ITT sites without a bias towards the GC base composition.

## Results

**Figure 1:**
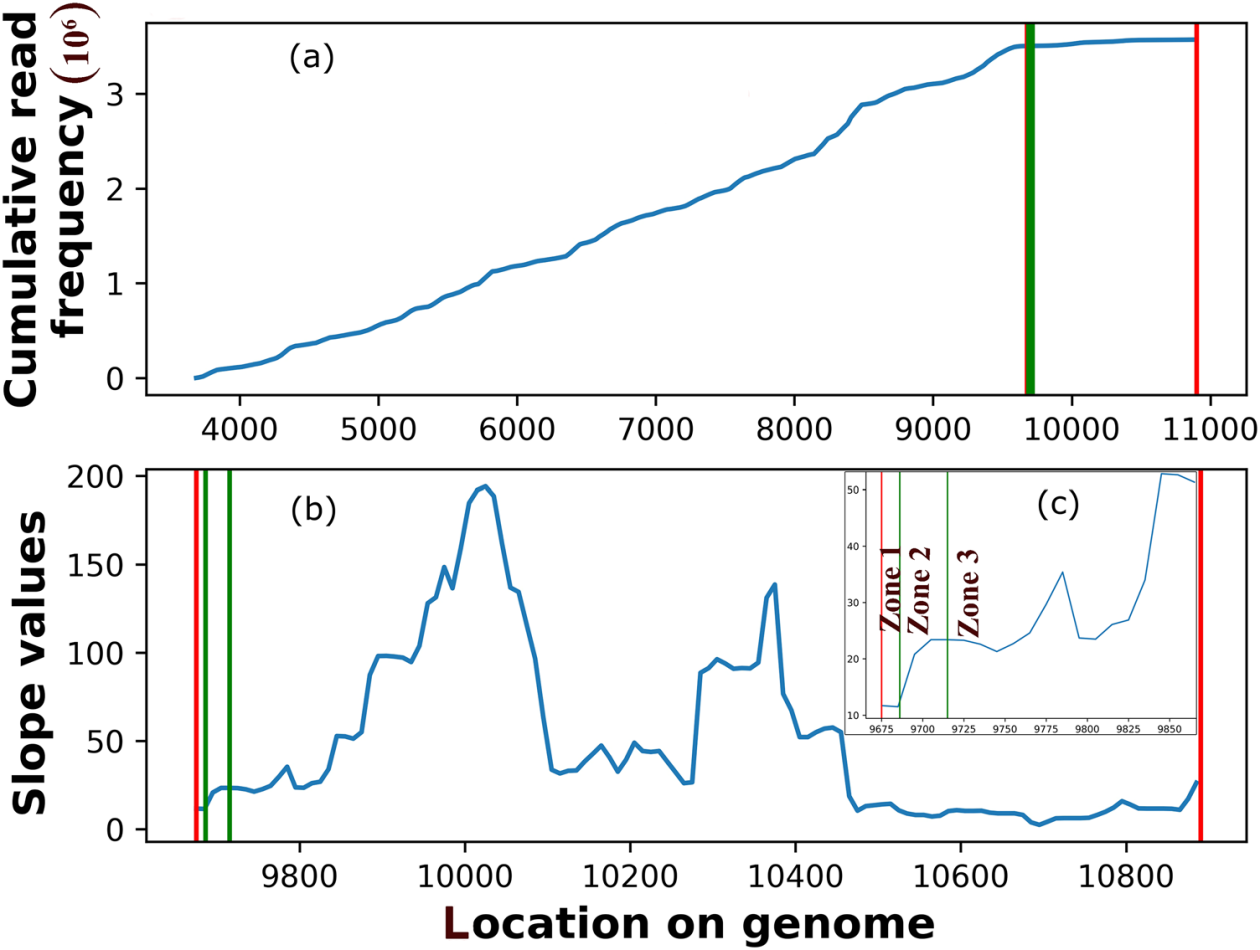
Defining IR and the positive slope region for screening a termination site. (**a**) A plot showing the cumulative distribution of the reads in and around an IR in *S. aureus* Newman, NCBI ID: NC_009641, RNA-seq ID: ERR1337989 for operon: [3686,9675,10900], where 3686–9675 is the coding region and 9675–10,900 is the IR marked by red lines. The blue curve shows the cumulative frequency of RNA-seq reads at each position in the above-mentioned operon. The numbers on the x-axis represent the actual genome coordinates. (**b**) The IR region for operon where slopes have been calculated from cumulative frequency values in **a**) using a window of 10 bases. A region of positive slope implies an increase in reads frequency after the previous drop. Red lines approximately mark the start and end of the IR (IR^start^ and IR^end^). The green lines mark the start and end of the first positive slope region (pos^st^ and pos^end^, respectively) of the curve, indicating the end of transcription should be before this first read-increase region. (**c**) The inset around the transcription termination region is shown schematically divided into three zones; Zone 1: stop codon to start of the positive slope region; Zone 2: start of the positive slope region to end of the positive slope region; Zone 3: end of the positive slope region to the end of the IR.

We look for the presence of an ITT site in the 13 bacterial genomes (Table 1). Using the Interoperonic Region (IR; Fig. 1a) as the scan window, we individually identify a hairpin in either *cluster* or *single*, with the presence/absence of poly U/A stretch. IR is defined as the region between the 3′ end of the last coding gene and the 5′ end of the first gene of the next operon. In the beginning, we identify 31,078 IRs in our dataset almost equally divided between the forward and the reverse strand (Table S2). Our data set includes both GC and AT rich genomes with the largest number (5065) of IRs from *Nostoc punctiforme PCC 73102* and least from *Fusobacterium nucleatum ATCC 25586* (885). On imposing the high confidence criterion of > 90% read coverage in an IR, we obtain 16,027 IRs (i.e., 52% of the starting data set). On further removing the IRs with outlier (5% tail of coverage-data) read depth, we obtained 14,738 IRs. *Nostoc punctiforme PCC 73102* and *Fusobacterium nucleatum ATCC 25586* have the lowest and the highest number of genes per operon, respectively (Table S3). We found the *RNA-seq derived* transcription termination site using a positive slope region of read depths (Fig. 1b) in 97% IRs; i.e., in 14,269 cases. Herein it is essentially the first region after the stop codon where there is a continuous increase in read frequency after a saddle point. This region is believed to indicate the terminal segment of the transcription unit. For 11,914 IRs (excluding Zone 3 cases; see “Methods”), we predicted hairpin(s) as well as a positive slope region (pos; see “Methods”), and thus these cases were used for validating our results. All alphabetical labels in diagrams and tables corresponding to the genomes are as per the list in Table 1.

**Table 1:** Organism list used in our study.

	Organism name	Bacterial phyla	GC%	NCBI ID	RNA-seq ID
*A*	Staphylococcus aureus Newman	Firmicutes	32.9	NC 009641	ERR1337989-91
*B*	Clostridium phytofermentans		35.4	NC 010001	ERR1709598
*C*	Myobacterium gilvum	Actinobacteria	67.9	NC 009338	SRR748310
*D*	Bifidobacterium pseudocatenulatum		56.4	AP012330	ERR2789029-30
*E*	Treponema denticola	Spirochaetes	37.9	NC 002967	ERR2177101-02
*F*	Leptospira interrogans		34.9	LT962963	SRR3222245-46, 49
*G*	Fusobacterium nucleatum	Fusobacteria	27.2	NC 003454	ERR2177097
*H*	Nostoc punctiforme	Cyanobacteria	41.4	NC 010628	SRR5197990
*I*	Prochloroccus. marinus		50.7	NC 005071	ERR2137696-98
*J*	Nodularia spumigena		41.3	CP020114	SRR5801900-01
*K*	Pseudomonas aeruginosa	Proteobacteria	66.6	NC 002516	SRR4237919-20
*L*	Syntrophus aciditrophicus		51.5	NC 007759	SRR3944268
*M*	Klebsiella pneumoniae		57.45	CP011985	SRR3018810,12

List of phyla and organisms used for validation of our method.

### Zonal demarcation of *RNA-seq derived* termination regions

**Figure 2:**
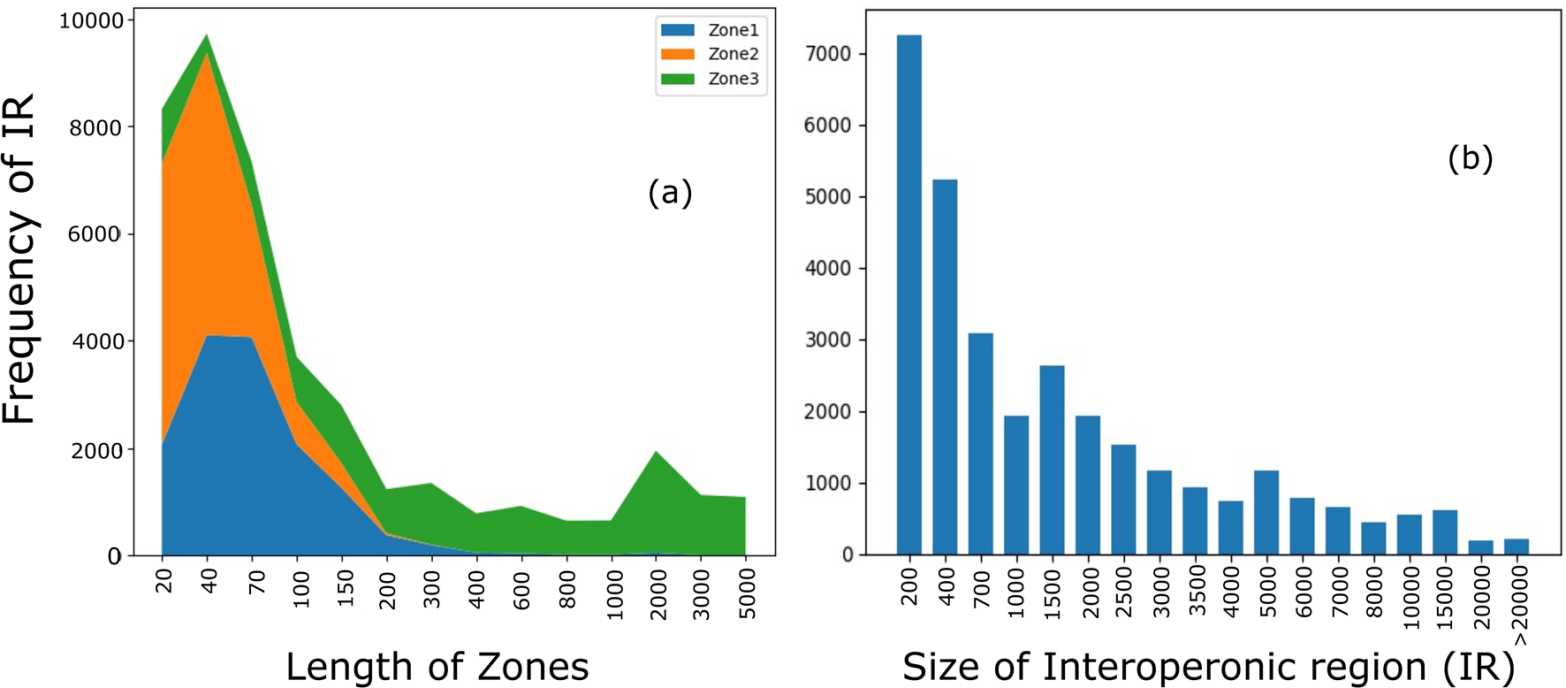
Distribution of Zone and IR lengths. Stacked line plot (**a**) showing the distribution of zone lengths for all genomes analyzed. The total number of IRs for which these zones have been calculated for each organism A–M as in Table 1 is as follows: 671, 991, 1135, 364, 455, 1090, 293, 1196, 646, 1491, 1378, 773, 631. (**b**) The distribution of IR sizes across all bacterial genomes studied. Note the difference in the Zone lengths and the IR lengths.

The zones of an IR are defined once the termination site is derived from the RNA-seq data using a positive slope region of read depths (see “Methods”). Zone 1: from the stop codon to the start of the positive slope region; Zone 2: from the start of the positive slope region to the end of the positive slope region; Zone 3: from the end of the positive slope region to the end of the IR. The demarcations are shown in Fig. 1c. They allow us to indicate the putative region where the transcription unit ends. Zone 1 is the end of the transcription unit; Zone 2 is the buffer region of the transcription end; Zone 3 is the likely beginning region for the next gene. In rare cases, the ITT may spill over to the initial region of Zone 3, but not further. The reason we call Zone 2 as the buffer region is that it straddles the entire positive slope region, which means that there is an increase of read occurrence in this region, indicating that ITT events are not anchored to a single fixed site in the genome, but span a broader region. The mean length in bases of Zones 1, 2, and 3 segmenting the IRs is found to be 62(± 17), 38(± 4), and 1617(± 194), respectively. The distribution for zone lengths across all genomes studied has been shown in Fig. 2a and Table S4. The average length of the Zones is determined by the average pos^st^, pos^end^, and length of the IRs in each genome. Pos^st^ is the start of the positive slope region, which marks the region of approximate constant RNA-seq read frequency in IR relative to the coding region. Pos^end^ marks its end. These positions are shown with green lines in Fig. 1. Out of 14,269 IRs where a positive slope region was found, 43% IRs have Zone 1 length less than 40 bases, another 43% within 100 bases, followed by 11% between 100 and 200 bases and extending up to as long as 1600 bases in the remaining 3% cases. The distribution of IR lengths can be found in Fig. 2b. A short Zone 1 indicates early termination, and an overwhelming 86% of cases terminate within 100 bases as per the RNA-seq data. About 96% of the Zone 2 lengths are less than 100 bases, with approximately 74% within 40 bases. In contrast, only 35% of Zone 3 lengths fall within 200 bases and 84% within 3000 bases, with 16% with even higher lengths. Short (< 100) Zone 2 lengths may indicate a tight buffer region for ITT, cognizant of the fact that the estimates may vary due to the window size for calculation chosen (see “Methods”). Long Zone 3 lengths suggest their suitability as sites for transcription initiation for the adjacent transcription unit.

### Hairpin prediction

As already outlined before, we term a group of hairpins such that the distance between the end of one hairpin and the start of the next hairpin is always < 15 nucleotides as a *cluster*, and others as *single.* A total of 677,943 stable hairpins is predicted for 87% of 14,738 IRs in our data set. The predicted hairpins form 131,610 *cluster*-hairpin (*cluster*) units giving an average of about 5 hairpins per *cluster*. The remaining 25,696 hairpins do not form any *cluster* and are deemed as *single*-hairpin (*single*) units. The number of hairpins is not proportional to the number of IRs, and the highest is predicted for *Mycobacterium gilvum* (at 161 hairpins / IR) followed by *Pseudomonas aeruginosa* (129 hairpins / IR). In comparison, the lowest is found in *Fusobacteruim nucleatum* with 15 hairpins / IR. The number of hairpins per IR (hairpin density) is a consequence of the GC percent of the IRs; the correlation coefficient is 0.8, which is expected due to the dominant contribution of GC hydrogen bonds to stem-stability in hairpins.

### Identified termination units

The *cluster* or *single* unit nearest to the stop codon is termed the *identified* termination unit since it is expected to provide the most efficient termination. It would also kinetically be the first hairpin to form and disrupt RNAP movement. A total of 12,787 termination sites (in 87% IRs) are identified closest to the stop codon, of which 7394 and 5393 could be linked to *cluster-* and *single-hairpin* units, respectively. The total number of hairpins in *identified clusters* is 22,519, with an average of 3 hairpins per *cluster*. We also calculated the *identified* termination units for rRNA (Supplementary Fig. S1) and tRNA genes (Fig. S2), but the focus in this work is only on protein-coding genes.

### Stem and loop characteristics

About 84% of all *identified* hairpins have 4–10 stem lengths, while 81% have 5–8 loop lengths. The < 14 bases and < 13 bases corresponding to one strand of the stem and loop length, respectively, covers > 90% of the *identified* hairpins. The mode of the distribution is contributed by 25% of cases having 4–8 stem and 6 loop lengths. The minimum stem and loop lengths are 4 and 5 bases, respectively. Overall, the loop length distribution of the hairpins in a *cluster* and *single* are almost identical, but the stem length distribution differs. For *cluster* units, 84% of the cases have a stem length of ≤ 10 bases; whereas, for *single,* this is 79%. The stem length of 10–20 occurs 5% more in *single* than *cluster* units. While these differences are not that large, it perhaps indicates a functional requirement for ITT. We also see that shorter stem hairpins are present at initial positions in a *cluster* (Fig. S3).

### Hairpins are found close to the stop codon

**Figure 3:**
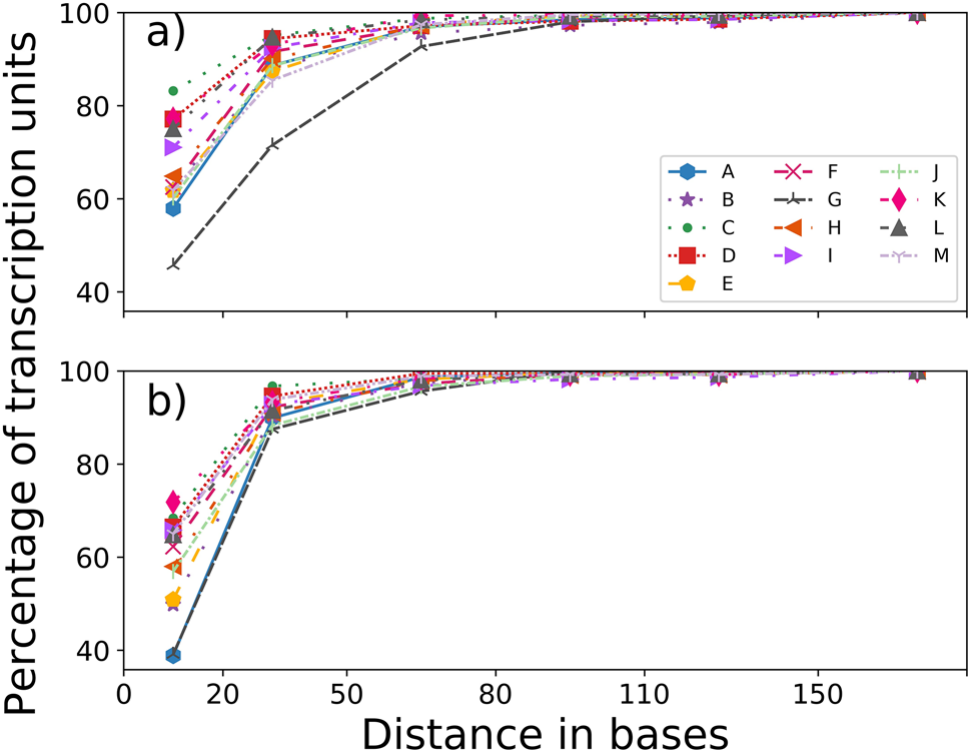
Distance of *identified* hairpins from stop codon. Plots show the percentage of transcription units with *identified* hairpins (y-axis) at a given distance from the stop codon (x-axis) in (**a**) *cluster* hairpin and (**b**) *single* hairpin. The organisms A–M are labeled as in Table 1.

We found 66(± 10)% and 58(± 11)% of the *identified cluster* and *single* units to be located ≤ 20 bases from the stop codon in all IRs (Fig. 3); for a window size of ≤ 50 bases, these numbers increase to 89(± 6)% and 92(± 3)%. Further extending the window (≤ 80) covers > 97(± 2)% of both *identified clusters* and *single* units in all organisms. Only < 2% termination units are present further up to 150 bases, and a negligible fraction beyond. A comparison of the total number of IRs for each genome indicates that, in general, a larger number of IRs has *cluster* units lying nearest to the stop codon than the *single* units (Table S5). Table 2 shows that in most organisms, the number of *identified cluster* units is greater than *identified single* units, but when this is not the case, the difference between their positions is not too large. This is not true in cases of *identified single* units that have a higher frequency, where we see a larger difference in distances. There is a 0.74 correlation of this difference with the GC content of the bacterial genomes analyzed. This value shows how strongly the GC content is correlated to favoring the *cluster* over *single* hairpin units. It can be inferred that a genome with high GC content is bound to have a higher number of the *cluster* as compared to *single* hairpin units.

**Table 2:** *Identified* termination units.

Organism	*Identified*	*Cluster*	*Single*
S. aureus Newman	719	264	455
C. phytofermentans ISDg	1076	476	600
M. gilvum PYR-GCK	1187	880	307
B. pseudocatenulatum DSM 20,438	399	214	185
T. denticola ATCC 35,405	503	277	226
L. interrogans Manilae L495	1162	656	506
F. nucleatum ATCC 25,586	335	151	184
N. punctiforme PCC 73,102	2124	1115	1009
P. marinus MIT 9313	685	463	222
N. spumigena UHCC 0039	1621	876	745
P. aeruginosa PAO1	1439	1052	387
S. aciditrophicus SB	816	509	307
K. pneumoniae UHKPC07	721	461	260

### Locating the RNA-seq derived termination units

The *cluster* or *single* units that are closest to pos^st^ are termed as *RNA-seq derived hairpins.* The distribution of *cluster* or *single* termination units in the forward and reverse strand and their zonal location is shown in Table 3. It is observed that the maximum number of termination units lies in Zone 1, followed by Zone 2 and a few in Zone 3 (data not shown for Zone 3). Of the 11,914 IRs for which *RNA-seq derived* termination units were found, 75% lay in Zone 1 and the remaining 25% in the buffer Zone 2.

**Table 3:** *RNA-seq derived* termination units.

(± Strand) hairpin	Zone 1	Zone 2	Total
(+ Strand) *Cluster*	3194	774	3968
(+ Strand) *Single*	1262	679	1941
(- Strand) *Cluster*	3221	779	4000
(- Strand) *Single*	1245	760	2005

*RNA-seq derived* termination units (excluding Zone 3) and their strand-wise zonal classification for each genome.

**Figure 4:**
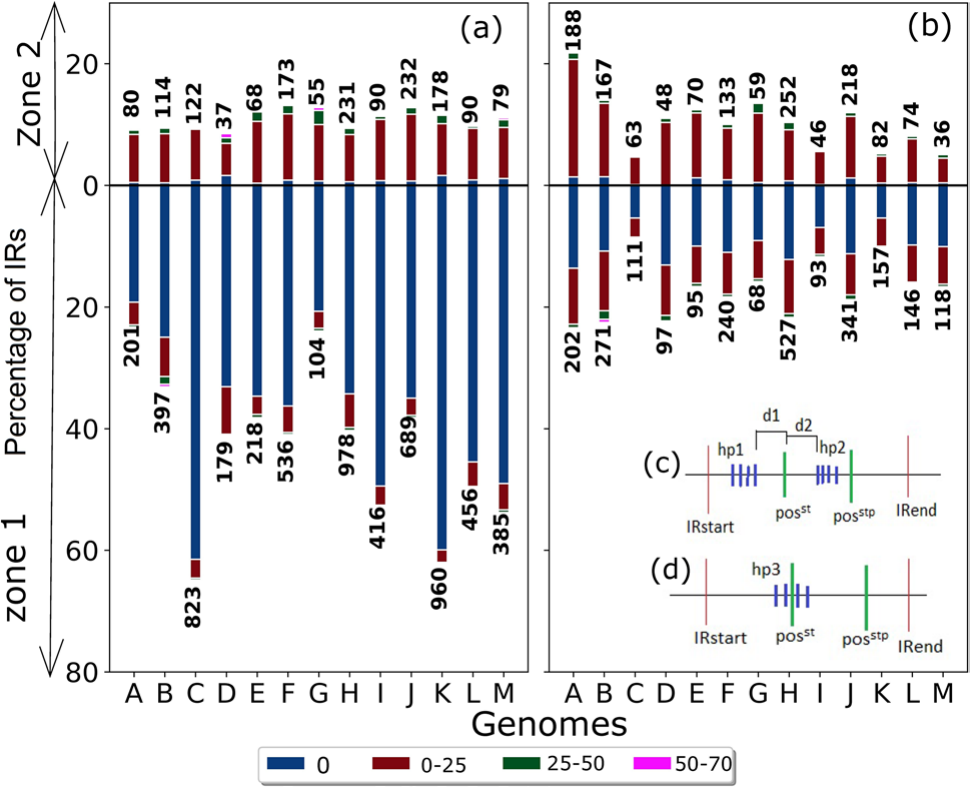
Zonal distance distribution of *RNA-seq derived cluster* and *single* hairpin units*.* Bars show the distribution of *RNA-seq derived* hairpin units with respect to distance from pos^st^ for the *cluster* (**a**) and *single* (**b**) units as per in zone location. The different window sizes (represented by different colors) show the different distances from pos^st^ and their corresponding percentages on the y-axis. The total IRs from which percentage has been calculated for each of the genomes A–M on the x-axis are given on top of each bar for Zone 2 and at bottom of each bar for Zone 1. The x-axis labels show the 13 analyzed genomes, as indicated in Table 1. The total number of hairpins contributing to each bar is shown on the top/bottom of each bar. There are 111 transcription units where the hairpin is located > 75 bases from the pos^st^; these are not shown in the plot. (**c**) For shortest distance calculation, if hairpin (hp) is in Zone 1 (between IR start and pos^st^), then n is calculated as the distance between the last hairpin in *cluster* unit (last blue line in hp1 *cluster*) to pos^st^. So d1 in the image is ‘n’ for zone 1 hairpins. If hairpin in Zone 2 (between pos^st^ and pos^stp^), then n is calculated as the distance between pos^st^ and the first hairpin of *cluster* unit (first blue line in hp2 *cluster*). So d2 in the image is ‘n’ for Zone 2 hairpins. (**d**) The *cluster* hairpin hp3 lies in Zone 1 and Zone 2. In such a case, we assign n = 0.

Figure 4 shows the IRs where the hairpins are found within window size ’n’ from the pos^st^ with ’n’ ranging from 0 to 75 bases. n is calculated as the shortest distance from the hairpin base, and therefore it is the last hairpin in the *cluster* unit for Zone 1 and the first hairpin for Zone 2. Figure 4c,d shows the calculation of the shortest distance in both Zone 1 and Zone 2. On average, 82% of hairpins in IRs where pos was detected, lie within 75 bases of the pos^st^. Of these, in 50(± 11)% cases, the pos^st^ is with n = 0; i.e., it coincides with the hairpin start. There is a gradual decrease of cases by distance with 29(± 6)%, 3(± 1)%, and 0.4% of the termination sites lying within 25, 25–50, and 50–75 bases of pos^st^. 13(± 1)%, 1(± 1)%, 0.3% of *cluster* units lie within 25, 25–50, and 50–75 bases, respectively. The corresponding values for *single* are 16(± 6)%, 1%, and 0.2%. Of the *RNA-seq derived* termination units, 67%, and 33% are from the *cluster* and *single*, respectively; therefore, higher occurrence of the former points to their utility in bacterial ITT.

More than 70% of transcription units have Zone 1 lengths ≤ 70, with a maximum of around 200 (Fig. S4). This means that the most calculated pos^st^ lies close to the stop codon, which indicates an early termination and correct estimate of the pos and the predicted hairpin. The Zone 2 lengths are largely restricted within 100 bases for all IRs (Fig. S5).

Energy score is the value obtained by MFold prediction (see “Methods”) that gives an estimate of the RNA secondary structure stability. This value can be used to compare the relative stability of hairpins. Looking at the energy score distribution of *RNA-seq derived* hairpin units covering Zone 1 and 2, we find that both zones together has 56(± 8)% hairpin units with energy scores between 0 and − 5 kcal/mole, 36(± 6)% between 5 and 0 kcal/mol and 7(± 2)% between − 5 and − 10 kcal/mol. A minuscule 1% fraction of hairpins has an energy score lower than − 10 kcal/mol (Fig. S6). All hairpins with 5–0 kcal/mol scores are also structurally stable, as confirmed from three-dimensional structure modeling studies (see “Methods”). The distribution of the energy scores across the two Zones is similar, except that Zone 1 tends to have a marginally greater proportion of lower energy scores. Only *T. denticola*, *P. aeruginosa,* and *K. pneumoniae* tend to have hairpin energy scores < −10 kcal/mol.

### Co-locating *identified* and *RNA-seq derived* termination units

**Figure 5:**
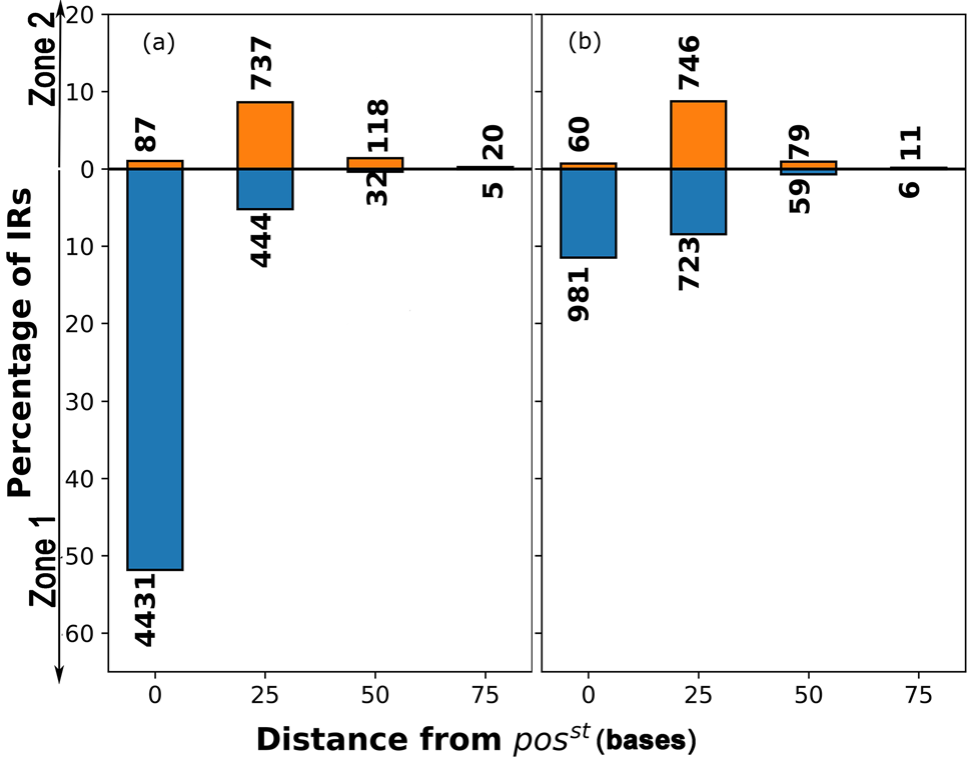
*Matched* termination units vs distance from pos^st^. Bar graph showing the percentage of *matched* termination units located within a given distance of the detected positive slope region. The number of cases in each bin is given on top of the bar. (**a**) *Cluster* termination units, (**b**) *Single* termination units. Five termination units located > 75 bases from pos^st^, are excluded from the plot.

**Figure 6:**
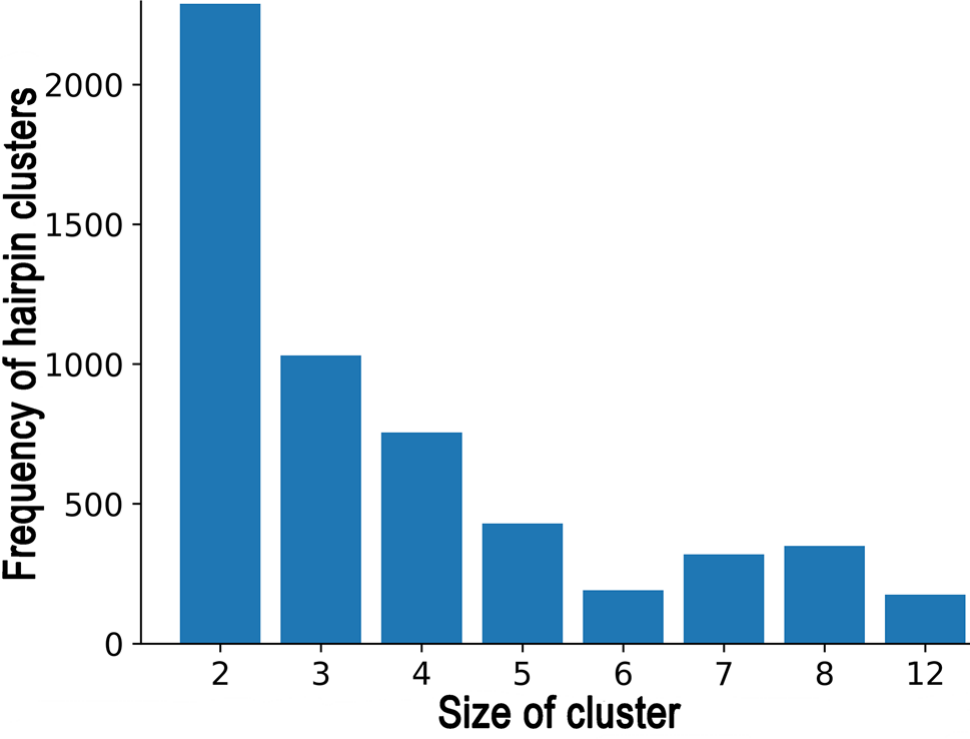
Size of *clusters*. The bar graph shows the frequency of hairpin *cluster* occurrence for different sizes of the *clusters*. The data shown here represents all *matched* termination units covering the 13 bacterial species studied.

An ITT site is expected to be close to the *pos*^*st*^ location. Since *RNA-seq derived* termination units are closest to *pos*^*st*^ and *identified* units are closest to the stop codon, we attempt to find co-locating hairpins between these two categories to validate an ITT. The co-located termination units are called the *matched* termination units. *Identified* and *RNA-seq derived* hairpin units occur in 87% and 81% of IRs, respectively, in the 13 bacteria studied (Table 4). For 11,914 IRs, where both *identified* and *RNA-seq derived* hairpin units are present, 72% occur at the same position (hairpins fully overlapped or partially overlapped). Not all organisms have a similar fraction of *identified* and *RNA-seq derived* hairpins found at the same location, with the best predictions for *P. marinus* at 83% and worst for *C. phytofermentans at* 46%. If we look at the distance of the *matched* termination units from the stop codon, 67(± 10)% of the *cluster*- and 57(± 11)% of *single* are within 20 bases (Fig. S7); correspondingly 90(± 6)% and 92(± 3)% within 50 bases, and < 1% cases located > 80 bases from the stop codon. Looking at the distance from pos^st^, 96% lie within 25 bases, of which 65% termination units embed the pos^st^ location (Fig. 5). Most importantly, 69% of the *matched* cases are from the *cluster*, while only 31% are *single* termination units. This corroborates the central role of the *cluster* units as the intrinsic terminators. Interestingly, 78% (57% *cluster,* 21% *single)* of the *matched* cases occur in Zone 1 compared to 22% (11% *cluster,* 11% *single*) in Zone 2. For remaining IRs, where *identified* and *RNA-seq derived* hairpin units did not match, 70% are within 60 bases of each other (Fig. S8). The two-hairpin termination units are the most prevalent cases, and their occurrence exponentially decreases with the increasing size of the *cluster* (Fig. 6). Interestingly, when large *identified clusters* extending beyond 100 bases are matched through large overlaps with *RNA-seq derived cluster* units, they highlight the importance of *cluster* hairpins in ITT (Fig. S9).

**Table 4:** Statistics showing the number of IRs matched with the RNA-seq data.

Genomes	Number of IRs^#^				Transcription termination site matching			
	Filtered		*Identified*	*RNA-seq derived*	Ahead*	Part-match	Full-match	% correct (Matched)
Staphylococcus aureus Newman	870		719	671	210	72	389	69
Clostridium phytofermentans	1259		1076	991	530	107	354	47
Myobacterium gilvum	1308		1187	1135	263	357	515	77
Bifidobacterium pseudocatenulatum	468		399	364	120	69	175	67
Treponema denticola	612		503	455	112	95	248	75
Leptospira interrogans	1338		1162	1090	285	220	585	74
Fusobacterium nucleatum	455		335	293	98	40	155	67
Nostoc punctiforme	2464		2124	1996	545	398	1053	73
Prochloroccus. marinus	803		685	646	108	172	366	83
Nodularia spumigena	1843		1621	1491	397	277	817	73
Pseudomonas aeruginosa	1585		1439	1378	329	409	640	76
Syntrophus aciditrophicus	955		816	773	161	221	391	79
Klebsiella pneumoniae	778		721	631	207	132	292	67
**Σ**	14,738		12,787	11,914	3365	2569	5980	72

^#^Filtered IR refers to those obtained after applying RNA-seq coverage and outlier-based filtering criteria; Identified IR refer to those where hairpins have been predicted by MFold program and lie closest to stop codon; *RNA-seq derived* IRs refer to those with hairpin located at the site in Zone 1 or Zone 2 and lie closest to pos^st^. *identified site is nearer to stop codon than *RNA-seq* *derived* site.

**Figure 7:**
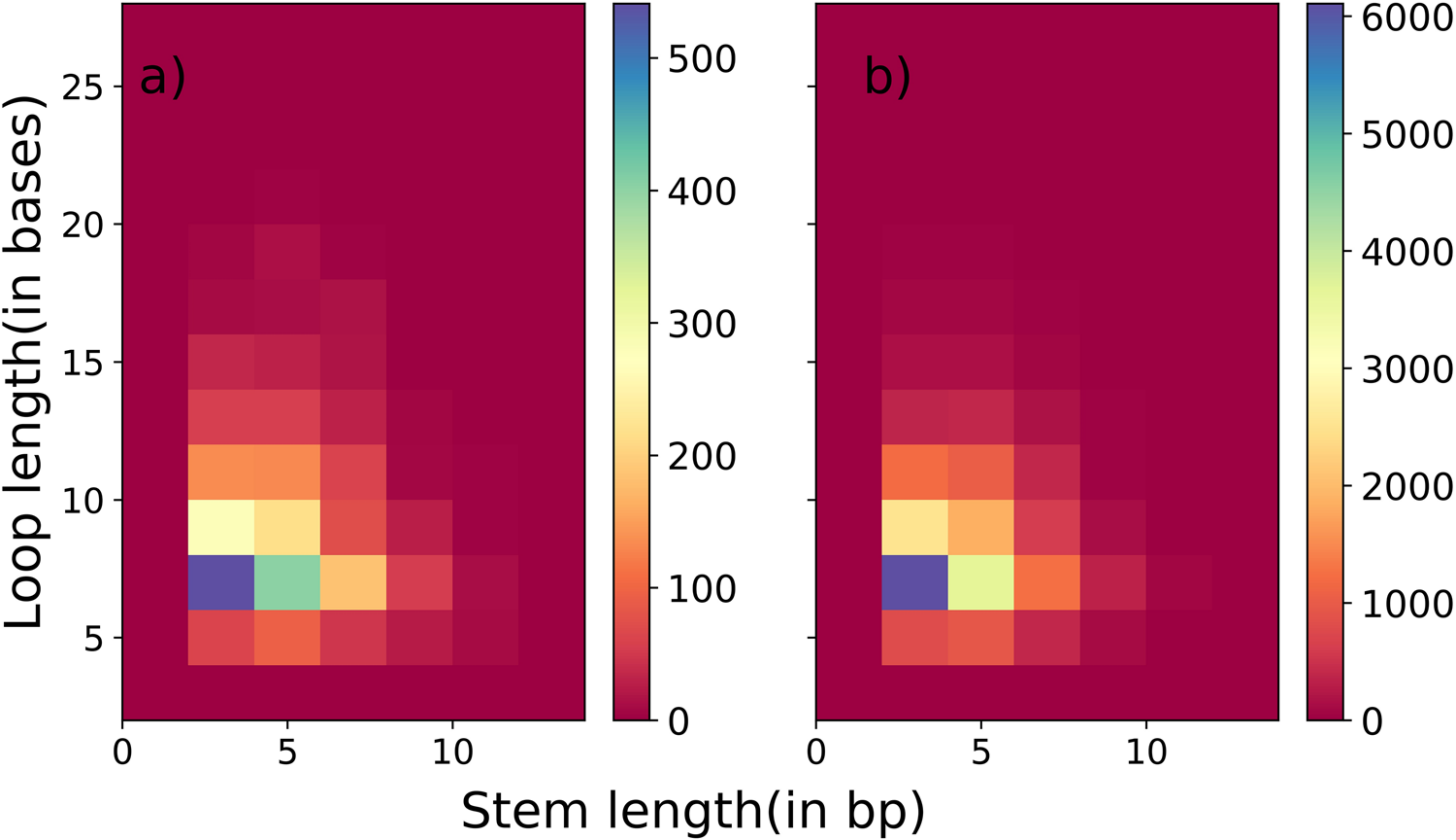
Plot showing the distribution of stem lengths (x-axis) and loop lengths for *matched* termination units in (**a**) *single* units (**b**) *cluster* units.

For *matched* termination units, the average stem length is 8 bp for *single* and 7.4 bp for *cluster* units, while the average loop length is 3.7 for *single* and 3.4 for *cluster* units (Fig. 7). Mode for stem length is 3 bp in *single* as well as *cluster* units, while for loop length it is 6 bases for both *single* and *cluster* units. The data indicates the predominant presence of short hairpins, which are kinetically faster to form. However, the length distribution of both stem and loop is right-skewed.

The *RNA-seq derived* site may not necessarily be at the same position as the first *identified* hairpin predicted by us but may match with other putative downstream termination sites. To check the same, we looked at alternate termination sites predicted by our algorithm for each operon. These are hairpins that are not the first occurring from stop codon but are identified as putative termination sites by the same slope finding method (see “Methods”). On taking all such predictions and matching the *RNA-seq derived* termination sites, we get a match of 97% cases overall (Table S6).

### Equal efficiency of hairpin identification in AT and GC rich genomes

Of the 13 genomes in our data set, we found six genomes to be GC rich with > 50% GC pairs. The number of hairpins and the *RNA-seq derived* GC content are poorly correlated, except when the number of hairpins increases to 9–16 per *cluster*. The observation points to local variation in the GC content of the IRs. Our method identifies hairpin units equally efficiently in GC as well as AT rich genomes. When the *identified*/*RNA-seq derived* hairpins are found co-located, they should be independent of the GC content, as evidenced by a low 0.22 correlation coefficient between the fraction of co-located units and the GC content of the GC-rich genomes. Likewise, the occurrence likelihood of stable hairpin decreases in AT-rich genomes. Therefore an equal efficiency in finding co-located units and the GC content in them is reflected from a higher 0.6 correlation value. We see that the GC % and occurrence of U trail are anti-correlated with a value of − 0.9. This is as expected because GC + A(U/T) content must add up to 100%.

### Presence of poly A/U trails is not necessary in genomes

**Figure 8:**
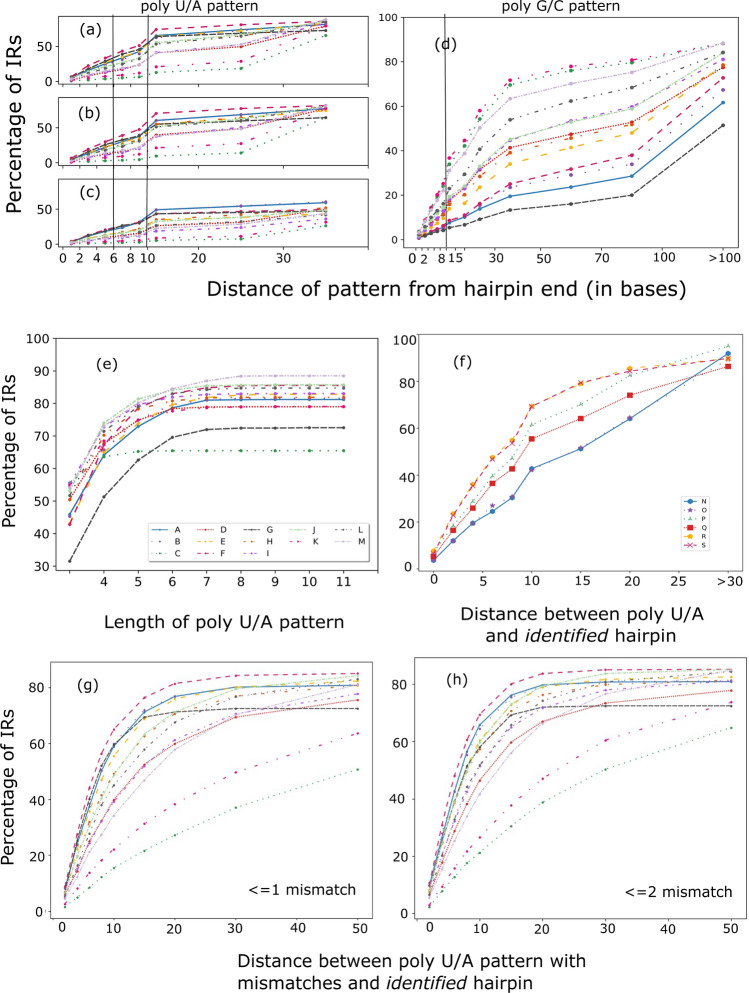
Line plots showing trends of poly U/A and G/C pattern occurrence in IRs. (**a**–**c**) Plots show the distribution of poly A/U patterns at a given distance between the hairpin end, where the first occurring poly U/A pattern has been considered. (**a**) depicts the statistics for all IRs where we have *identified* hairpins, (**b**) *cluster* + *single RNA-seq derived* hairpin units, (**c**) *single RNA-seq derived* hairpin units. Plot (**d**) shows the distance between *identified* hairpin units and the first occurring poly G/C pattern. Vertical lines at distances 6, 10 in (**a**–**c**)**,** and only 10 in (**d**) are drawn to highlight the low occurrence of patterns close to the hairpin end. Plot (**e**) shows the lengths of the first A/U patterns in all IRs. The different colors from (**a**–**e**) and in (**g**), (**h**) are the 13 bacterial genomes analyzed and follow the same order as in Table 1 and shown in legend with the plot (**e**). Plot (**f**) shows the distance between the hairpin and the first occurring pattern after them for *identified* hairpin units where genomes are N: *Escherichia coli* K-12 substr MG1655, O: *Escherichia coli* O127-H6 str. E2348, P: *Bacillus subtilis* subsp. subtilis delta6, Q: *Bacillus subtilis* subsp. subtilis N3-1, R: *Helicobacter pylori* PeCan4, S: *Helicobacter pylori* Lithuania75. Plots (**g**) and (**h**) show the same statistics as (**a**) where the poly A/U patterns have ≤ 1 or ≤ 2 mismatches, respectively.

We decided to look at the occurrence of poly U trail at the ends of *identified* termination units using the transcribed DNA information. Of the 13 diverse bacterial genomes scanned for A/U patterns, the existing patterns are rarely co-located at the base of the *RNA-seq derived* and *identified* hairpins (Fig. 8a–c, Table S7). The genomes show only 4% poly A/U patterns (at least three consecutive U/A) located within the first two bases of the *identified* hairpin; 12% within 4 bases, and 31% within 10 bases from the *identified* hairpin. Corresponding values for *RNA-seq derived* hairpins are 4%, 11%, and 29%. Only 4 genomes show > 40% poly A/U patterns within 10 bases of the hairpin. We see that the poly G/C pattern is also not present downstream of hairpins (Fig. 8d). We could find only 18% operons with downstream C/G pattern even at 15 bases from the hairpin end.

We additionally analyzed the well-studied *E. coli, B. subtilis,* and *H. pylori* genomes to check the occurrence of poly U patterns for all operons. We see that these organisms too follow the same trends as all other 13 genomes analyzed above. Even for *E. coli*, poly U trends were similar to other genomes in most operons (Fig. 8e).

We found that all organisms except *T. denticola*, *L. interrogans, F. nucleatum* had patterns of length 3 in 45% or more IRs with a total average of 49% (Fig. 8f). The larger > 6 poly A/U patterns are spread in 2% of all IRs, with about 20% IRs having no poly A/U pattern at all.

When allowing mismatches in the poly U/A pattern, as expected, the percentage of IRs with a poly U/A pattern at distances < 4 nucleotides from the *identified* hairpin end, increases from 12% for only perfect matches, to 18% with ≤ 1 mismatch and 21% with ≤ 2 mismatch, which is still low. For patterns at < 10 nucleotides from the *identified* hairpin end, the numbers change from 31% in perfect match to 45% with ≤ 1 mismatch and 52% with ≤ 2 mismatches (Fig. 8g,h). But this increase is organism-specific. With ≤ 2 mismatches, only 5 out of 13 organisms have at least 50% operons where a pattern was found within 8 bases from the *identified* hairpin end. So, it can be said that the poly U/A pattern is a feature that is used only by some organisms for ITT in an enhanced manner. To understand this trend, it is required to run this analysis on a larger dataset. But it can be seen clearly that poly U is not a necessary trait in the ITT mechanism for all bacteria.

### Comparison with in vitro screened experimental termination sites

A list of 143 experimental termination sites cumulated by Carafa et al.^13^ and tested in vitro for ITT activity was used to match our results. BLAST software (https://blast.ncbi.nlm.nih.gov) was used to search the sequences for their corresponding mapping regions in E. coli K-12 MG1655 (NCBI ID: NC_000913/U_00096) for which we also calculated the *identified* hairpins by our algorithm. It may be noted that Carafa et al. used a list of literature-identified hairpins that have poly U stretch with a strong bias of U to be present in the first two positions from the 3′ end of the hairpin. Also, these hairpins must have either 4 GC base pairs in the stem if no bulge or internal loop exists, or a minimum of 6GC base pairs if otherwise. Also a liberal criterion is used whereby termination zones are used to identify ITT location instead for of the exact location of the terminator hairpin. Yet we found only 83 of the 143 sequences to have a match in the IR region. Of these only 8 termination zones matched the *identified* hairpin or alternate termination site (other putative hairpins if the first site does not terminate transcription). In 3 other cases, we found a partial match between hairpins and the termination zones. On dividing the matches strandwise, 131 cases were found on the forward strand and remaining on the complementary strand. Of these, in 96 cases the hairpin was present either inside the operon region in the forward strand (36 cases, see hairpin 4 in Fig. S10) or the reverse strand (60 cases, see hairpin 2′ in Fig. S10). Other hairpins (see hairpins 2 and 3, Fig. S10) are present towards the end of the IR as opposed to our identified hairpins that lie close to the start of the IR. GC composition analysis showed only 47 of the 96 cases were GC rich (19 of 36 in forward and 28 of 60 in reverse strand after taking sequence complement). Therefore, the imposition of minimal GC base pairing criteria by Carafa et al. while selecting the hairpin sequences is not reflected in the GC composition of the hairpins as a whole. The mode value of 3 for stem length that we have obtained in our analysis of all matched hairpins is closer to the Carafa et al. minimal criteria, except that our hairpins also include AT-rich cases. To rationalize the poor match between the *identified* hairpin and the experimentally determined termination region, we tested if the *RNA-seq derived* termination sites overlap with these in vitro determined termination zones. In 8.4% of the 83 cases found in the IRs, the *RNA-seq derived* termination sites overlap, which is the first encountered positive slope region from the stop codon. But if we consider other downstream positive slope regions in the IR, we see that in 60% additional termination zones, the slope values either match to the region of *RNA-seq derived* site or are of a greater value. This reveals that most of the terminations zones are present very close to the end of the IR region, which would place them in Zone 3 as per our analysis methodology. As a result, they may be part of the 5′ UTR regions of the next operon suggesting that these may be in reality be a pause site or an attenuator/antiterminator^30^ in the in vivo situation (see hairpin 2, Fig. S10).

## Discussion

In bacteria, the process of transcription and translation is concurrent^31,32^ only for protein-coding genes/operons. Operating both processes in concert requires precise kinetic control of the mRNA produced and the amino acid translated. It has been seen that terminator sequences present in the coding region are not able to terminate transcripts. This is because the closely trailing ribosome does not allow the folding of sequences to a hairpin structure^31^. It is only after the occurrence of a stop codon that decoupling takes place and hairpin sequences can form their secondary structure to disrupt the RNAP motion.

The lack of solid evidence on the wide prevalence of ITT units across diverse organisms has, therefore, been intriguing. The extrinsic transcription termination mechanism involves concertedly acting protein factors and thus requires more sophisticated control than ITT. Therefore, our identification of 72% of the bacterial IRs with an ITT unit validated by RNA-seq (i.e., *matched* hairpins) read profiles bridges the conjecture that bacteria commonly prefer the ITT mechanism as their favored transcription regulation process. This inference is an improvement over our previous observation^18^, where we identified approximately one-third of the IRs to have stable hairpins based on GC-rich stem composition criteria.

For a basic ITT process to occur, the *single* hairpins are said to form at the rear end of the RNA exit channel^6^. For a hairpin to form, the RNA needs to fold while the transcription is occurring. Although previous studies proposed limits on hairpin lengths that can be accommodated near the RNA exit channel^6^ required for the formation of a functional hairpin^4,13^, these have been observed only in select organisms such as in *M. tuberculosis*^29^ and not across most bacteria. Our observation suggests that a stretch of contiguous hairpins (hairpin *cluster*) is as effective as a *single* strong hairpin to cause ITT. As such, most isolated hairpins can fold in solution within microseconds^33^ and the rate is determined by the number of nucleation points in the sequence^34^. For example, in hairpin helix, A_6_ C_6_ U_6_, at 24 °C the rates of formation and dissociation are 2.4 × 10^4^ s^−1^ and 2.6 × 10^4^ s^−1^, respectively^35^. Similarly, for 21 nucleotides hairpin UAUAUCGC7CGAUAUA at 37 °C, they are 1.3 × 10^6^ s^−1^ for the formation and 3.2 × 10^6^ s^−1^ for dissociation^36^. In contrast, the average mRNA elongation rate in *E. coli* is found to be 58 nucleotides/sec at 37 °C. Thus, the RNA folding rates are much faster compared to elongation rates as corroborated by experimental data^37^. The time difference is large enough to accommodate the actual folding time variations of a hairpin arising out of multiple factors including stem and loop sequence, neighboring bases, which alter the rate-limiting step of folding^38^. Folding experiments also show a preference for alternating structures (11 out of 14 to 40-length sequences) where there is interconversion between *single* and two hairpin states, the latter being the major one^34^. When long mRNA sequences fold it was observed that short-range interactions have higher stability over long-range reactions, and they are thus retained^39^. In physiological conditions, the number of accessible nucleation points and short-range forces are also expected to dominate the RNA folding process. However, folded hairpins herein may face a steep transition barrier to interconversion due to concomitant RNA–protein interaction; thus early formation of short hairpins forming a *cluster* would be preferred as suggested by our genome analysis.

Nascent hairpin formation during transcription may be impeded by spatial constraints in the RNA exit channel; however, during ITT, hairpins are known to form outside the channel^40–42^, once the loop-closing base pair is available. In in vivo situation, the RNA secondary structures may fold sequentially as the RNA gets elongated, but they rapidly change to alternating more stable structures^43^. This reduces the chance of misfolded structure formation, as observed in ribozyme folding studies^44^. However, small hairpins (like in *cluster*) may have similar folding rates both in vivo and in vitro as shown by hairpin folding studies of ribozymes^44^. Accessory factors like the NusA may further enhance the hairpin folding rates in vivo.

From structural studies, it is known that an RNA hairpin can pause transcription^45^ by stabilizing the RNAP structure by virtue of its interaction with the protein complex. Therefore, a *cluster* unit, which presents a larger count of shorter hairpins may further stabilize the RNAP complex by additional RNA–protein interaction, and this is expected to be more efficient in stalling and dislodging the transcription complex than a *single* hairpin. This is statistically consistent due to the higher occurrence of loop regions from shorter hairpins that can engage the protein in both hydrogen bonding and hydrophobic interactions. In such circumstances, many short contiguous hairpins can be more effective in RNAP pause and dismantling. It is also possible that the initial hairpins in the *cluster* slow down the polymerase while the later hairpins finally cause the release^21^.

It is interesting to note that the presence of secondary structures that hinder the formation of terminator hairpins reduces termination efficiency. This was shown by the application of force that suppressed the formation of such secondary structures, which led to an increase in termination efficiency^46^; therefore, our observation of the first hairpin (or *cluster* of hairpins) downstream of the stop codon as the effective terminator unit is consistent with the optimal arrangement necessary for efficient ITT. Forward translocation, another suggested mechanism of RNAP dissociation in ITT is also consistent with our proposition. Here the hairpin formation forces the RNAP to move downstream (relative to DNA template) while the mRNA does not move, thus breaking the DNA: RNA hybrid bonds. *Cluster* hairpins are dominantly placed to promote dissociation, reducing the over-reliance on poly U/A occurrence, which are thought to weaken the links in the DNA-RNA hybrid for easier dissociation.

The ITT, although perceived, is not entirely intrinsic to RNA, DNA, and RNAP activity alone, and is influenced by accessory factors like the NusA and NusG during transcription and termination^47,48^. These factors work by two modes during ITT, one by interacting with the RNAP and stabilizing it or interact with the emergent RNA transcript^6,42,49,50^. The NusA and NusG are known to form complexes to enhance their interaction with RNAP to increase termination efficiency; whereas, NusG alone is known to suppress transcription pause, but not termination^47^. Factors like RfaH, a homolog of NusG suppresses stabilizing effects of nascent RNA hairpin^51^ and decreases termination^52^. The interplay of accessory factors thus complicates the building of a true ITT thermodynamic model. Attempts for such a model two decades back have focussed primarily on understanding the stability of the termination complex, especially the contribution of RNA–DNA base-pairing^53,54^.

*Cluster* hairpins can, however, explain simple entropy-driven processes that may achieve efficient ITT. Such processes have been investigated in less complex systems for understanding the potential mechanisms at play^55,56^. If we assume RNAP:DNA:RNA hybrid and the immediate solvent environment as a quasi thermodynamic system inside the cell, the hybrid typically contributes to entropy change by its thermal fluctuations and translocation during transcription. The nucleotide addition cycle during transcription gradually alters the entropic state of the system till the time the nascent RNA at the exit channel is long enough to fold into a hairpin. We already know that RNA folding and thermal equilibration of the structure occur much faster than nucleotide addition by RNAP^37^. As RNA folding is driven by entropy^57^ there is a rapid entropy change locally in the system. This entropy change can act as a perturbation and a trigger for conformational transitions along soft modes^55^ of the RNAP complex. The perturbation may drive thermalization in the system leading to overcoming the transition barrier^56^ for ITT to take place. The probability of such perturbations increases with the formation of more hairpins and thus increases the probability of stochastic ITT. This predisposes *cluster* hairpins for more efficient ITT compared to the *single*. In vivo studies in *M. smegmatis* indeed show that two tandem hairpins downstream of the *gyrA* gene have a higher termination efficiency than individual *single* hairpins^21^. Please note that accessory factors that interact with the RNAP or the nascent RNA can alter the probabilities of the ITT process, but the proposition that *cluster* hairpins can achieve more efficient ITT than *single* hairpin remains consistent on both kinetic and thermodynamic considerations.

Termination takes place 10–14 nucleotides downstream from the first nucleation point of the hairpin^11^. This is consistent with our study as well. Another study found that termination (although not very efficient) was seen 7 nucleotides downstream from the stem end of hairpin^32^ in both in vitro and in vivo. There is also a relationship between the stop codon and the terminator position. Hairpins should be present at least 30 nucleotides away from the stop codon^31^. It has also been observed that terminators within 100 bases from the coding region have high termination efficiency. As mentioned above, all the analyses are performed only on the *E. coli* genome, and further experimental/mechanistic studies would be required to see if these trends are statistically significant and followed across other bacteria. We also tried to find regions of conservation near the stop codon and around the hairpins but could not find any such sequence conservation within 25 bases from the stop codon (Table S8; Fig. S11).

The other piece of the puzzle stems from the fact that extrinsic transcription termination also involves a hairpin upstream of the hexameric Rho protein binding site. Table 5 depicts data from literature where the concentration of RNAP subunits and other termination factors are expressed as a ratio of the Rho protein (i.e., < protein of interest > / < Rho protein >). Since the hexameric form of the protein is needed for RNA binding and extrinsic termination, for 100% extrinsic termination, we expect a 1/6 (0.167) abundance of RNAP. However, if we look at the maximum expression values for RNAP subunits for each experiment, the data suggest a low prevalence of extrinsic termination (approximately 10–25%). Given that the coverage of intrinsic terminators is in the range of 75–85%, together with the extrinsic terminator, it appears to cover the whole set of coding genes.

**Table 5:** Expression values of proteins as a ratio against Rho protein expression.

#	*E. coli*						*L. interrogans*				*M. tb*			*P.aeruginosa*		
	A	B	C	D	E	F	G	H	E	I	F	I	E	F	J	E
1	1	0.9	0.7	0.6	0.5	0.7	0.9	2.1	1.3	1.3	0.4	0.4	0.3	1.7	1.5	1.6
2	1.3	1.4	1.2	0.4	1	1	0.8	1.6	1	1.1	0.9	1	1.1	0.9	0.6	0.8
3	1.5	1.4	1.5	0.9	0.9	1.7	1.7	3.2	2.2	2.5	4	4.1	3.8	6.5	3.4	4.6
4	0.7	1	0.8	**0.3**	0.6	1.2	1	2.8	1.7	1.4	1.5	1.5	1.4	3.6	2.6	3
5	**0.5**	1.2	**0.3**	**0.4**	**0.2**	0.7	**0.4**	1.3	0.6	0.6	0.9	0.8	0.7	2	1.5	1.7

^**#**^1 = NusA, 2 = NusG, , 3 = RpoA, 4 = RpoB, 5 = RpoD; Methods: A/B/C^71^, D^72^, E^73^, F^74^, G/H^75^, I^76^, J^77^; values ≤ 0.5 are marked in bold for RNAP subunits, RpoA, RpoB and RpoD.

Existing literature suggests that Rho-dependent terminators mostly occur in enteric (rod-shaped gram-negative) bacteria^5^. Rho protein has been found to be inessential in many gram-positive bacteria, including *B. subtilis* and *H. pylori* species^22,58,59^. Also, the inhibition of Rho protein in these species has been linked to the non-essentiality of Rho^60^. Likewise, Clarke et al.^61^ have shown that the strong Rho-independent terminator sequences show the same conserved effects on RNAP activity across domains of life. Different tools that identify hairpins like Transterm^15^, RNAMotif^16^, etc. have similarly applied the search for these common features on bacterial datasets. It has also been seen that sometimes an operon has both termination sites (Rho-independent and Rho-dependent) as a fail-safe mechanism for termination^32^, but they work independently. The insights from our work appear to be consistent with these previous observations.

Other functional roles of hairpins might involve the presence of specific sequence motifs, or in inhibition of RNA degradation^49^, as DNA uptake elements^62^, translational surveillance^63^, or riboswitches^64^. However, the location of these elements is distinct from the region downstream of the stop codon.

To summarize, we identified 72% of cases of the *cluster* and *single* units in filtered IRs co-located against *RNA-seq derived* terminators. We show that if hairpins work in tandem (i.e., *cluster)*, they can bring about ITT, as evidenced by the absence of RNA-seq reads contiguous to our *identified* hairpin sites. 91% of the *matched* bacterial termination units are present close to the stop codon in each transcription unit within 50 bases. They typically have a stem length between 4 and 14 base pairs and a 5 and 10 loop length. The requirement of a poly A/U pattern at the hairpin base is not essential. The detection of hairpins is independent of its GC content; i.e., we are able to find ITT units across GC as well as AT rich genomes. Our work contributes to the understanding of the genomic requirements necessary to execute an ITT process.

## Methods

### Test data set

**Figure 9:**
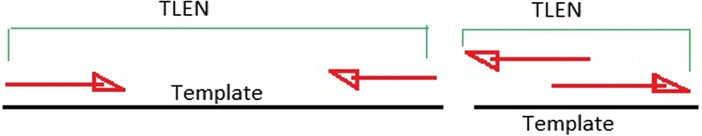
TLEN calculation. The illustration shows how TLEN is calculated for paired-end reads. The definition from the bam specification says that TLEN or the signed observed template length is the length between the mapped end of the template to the mapped start of the template for all reads mapped to the same template (https://samtools.github.io/hts-specs/SAMv1.pdf). This means the distance between the 5′ end of the leftmost read and the 3′ end of the rightmost read is mapped to the template.

A dataset is constructed from representative microbial species selected from 6 different bacterial phyla. At first, all organisms are grouped based on their phylogenetic distance from each other, as proposed by Ciccarelli et al.^65^. A representative from each group is chosen if it had public RNA-seq data (Fig. S12, Table 1) with ≥ 70% alignment with its reference genome (i.e. ≥ 70% reads mapped). All data is obtained from the NCBI-SRA database (http://www.ncbi.nlm.nih.gov/sra). Entries from Planctomyces, Thermodesulfobacteria, Chlamydia, Chloroflexi, and Acidobacteria from bacterial phyla are excluded due to the absence of high-quality RNA-seq data. To increase the confidence and coverage of RNA-seq data being used, sample replicates under the same experimental conditions are taken wherever available. This data is indexed and aligned to the respective reference genomes using Bowtie2^66^. Reads present in a proper pair (flags: 83/163, 99/147), with Transcript Length (TLEN) or insert size ≥ read length, and map quality ≥ 30, are filtered out from the initially obtained alignment. TLEN is the distance between the 5′ end of the leftmost read to the 3′ end of the rightmost read that is mapped to the same cDNA fragment (Fig. 9). Reads on the forward and reverse strand are also separated to get correct transcription levels for each transcription unit.

### Predicting hairpins

MFold^67^ software is used to predict the secondary structures for the input single-stranded RNA sequences. It has been used widely and tested for performance by several groups^68,69^. In MFold each gene and the corresponding RNA substring from the region of − 20 to + 270 bp is taken for analysis, where + 1 denotes the stop codon site^18^. MFold calculates free energy for predicted structures at 37 °C by assuming a two-state model^70^. The hairpin stability is calculated by penalizing various factors like length and sequence of the loop, stacking of the first mismatch from the initiation free energy, ∆*G*_initiation_^71^. Bonuses are added for special loop closing groups like GU. Output scores denominated as ∆*G* (kcal/mol) represent the stability of the hairpins. The scores in terms of ∆*G* output by MFold represent the stability accrued primarily from the secondary structure interactions. Additional stability may accrue from tertiary structure interaction, which may not be faithfully accounted for by the MFold scoring function. To check for this fact, we took all the predicted hairpins with > 0 ∆*G* (kcal/mol) score from MFold and subjected them to three-dimensional structure modeling using SimRNA^72^. SimRNA is a computational RNA three-dimensional structure prediction method that uses Monte Carlo simulations to check possible folding structures and outputs an energy score for it. The secondary structure output from MFold was used to put constraints on the conformational search space of the three-dimensional structures during sampling. For each hairpin, we find 11 all-atom structure frames after 1600 iterations and add protons using the REDUCE program^73^. We screen if the predicted three-dimensional RNA structure is valid with help of the MolProbity program^74^. It checks for the presence of wrong sugar puckers in each predicted structure. Trial three-dimensional hairpin models are first generated in batches to check for stable structures. All the hairpins returned at least one tertiary model with < 0 ∆*G* (kcal/mol) score among the first 100 trial structures generated respecting the expected sugar puckering geometries, bond length and bond angle values. To additionally confirm the veracity of our methodology, we searched through the literature and culled 110 hairpin structures for which we had information on experimentally determined free energy of hairpin formation. These hairpins have been predominantly reported in *E. coli*, in addition to *P. aeruginosa*, *M. formicicum*, and *T. acidophilum*^70,71,75^. The sequences are subjected to MFold secondary structure prediction, and those with > 0 ∆*G* score are taken for tertiary structure modelling using SimRNA as outlined above. All the energy scores output for the three-dimensional models are found to be < 0 ∆*G* (kcal/mol) (Fig. S13). The predicted hairpins in our study had a wide range of stem and loop lengths and this was consistent with the previous reports that corroborate the absence of any correlation between hairpin strength, stem or loop length^76^. Our results indicate that all MFold predicted hairpins are stable and thus taken forward in our analysis. For each MFold output, hairpin structures are extracted and labeled as *predicted hairpins*. A two-dimensional histogram is used to find the counts of various combinations of stem and loop lengths for the predicted hairpins. The lowermost counts for the combinations constituting 5% of the total cases are rejected retaining only those hairpin loop-stem length combinations satisfying the threshold. This was done to exclude potential outlier cases.

### Defining the transcription termination scan window

RNA-seq reads do not reduce in number in the intergenic regions of an operon^77^; therefore, the non-coding region downstream of a stop codon of an operon or individual gene is found for each transcription unit up to the defined scan window (+ 270). The FGENESB module of the Molquest software (http://www.molquest.com)^78^ is used to obtain the start and stop codon positions of genes and operons. It predicts operon locations using distances between locations of predicted genes in combination with data from homolog databases like COG^79^, KEGG^80^ after performing a BLAST^81^ search. It uses a Markov model for coding region annotation, and distances between predicted genes to predict operons. It supposedly predicts 70% single transcription units and about 50% operons exactly. Accuracy determination is done on a set of difficult short genes that have been used for evaluation accuracy of bacterial gene finders as well. It was seen that the accuracy of Molquest is better than previously used Glimmer and GeneMarkS softwares^82^, which previously provided the best accuracy. Accuracy was tested on highly annotated sets from *B. subtilis* and *E. coli*. Predicted promoters and terminators are used to increase the accuracy of operon identification, as they now have defined boundaries thus training of initial parameters is better. Genes annotated in the NCBI genome database but not identified by Molquest are included in our list as additional entries. We calculate the inter operon/gene region (IR), defined by the region between the 3′ end of an operon or individually transcribed gene and the 5′ end of the next transcription unit. The intergenic regions within an operon are not included in IR.

### IR screening by RNA-seq data

For each genome, read depth is calculated at each location. In case the organism had replicate experimental data, reads from all of them are combined and averaged to find an average read depth. Only cases where the read coverage of the CDS region is > 90% are taken for further analysis because of PCR amplification errors, degree of annotation in operons, PCR duplicates, alignment efficiency of paired-end mapping, etc.^83^. If read coverage in either the CDS or IR region of a transcription unit is several orders higher than the rest of the genome, it is removed as an outlier; for this, we check the 5% tail region of the average read depth histogram of CDS or IR regions.

### Defining termination units

Out of all predicted hairpin structures, those falling within screened IRs are filtered out for the two strands. These structures belong to a *cluster* if the distance between the end of one hairpin and the start of the next is < 15 nucleotides. All such clusters are labeled *cluster*. In the case of larger hairpin subsuming the smaller, first occurring (i.e., the larger) hairpin is chosen for *cluster* calculation. The choice of < 15 nucleotides as hairpin separating distance for identifying hairpin *cluster* is based on the typical length of the nascent RNA nucleotide strand that can be enclosed in the RNA exit channel (5 nucleotides) and DNA-RNA hybrid (9–10 bp)^6^ and unavailable for folding into a hairpin at any given time. It is ensured that a subset of hairpins from a higher size *cluster* is not present as a separate entry in a lower size *cluster*. Aside from the *cluster*, those hairpins without any neighbor at < 15 nucleotides are labeled as *single*. For those cases where two hairpins have partial overlaps, each is checked if they can form a *cluster* as per the defined criteria or left as a *single* hairpin. We take each *single* or *cluster* as a potential termination unit, irrespective of the size of the hairpin or the number of hairpins in a *cluster*. For each IR, such a termination unit lying closest to the stop codon is labeled as an *identified cluster* or *identified single*.

### Identifying the termination region by RNA-seq data

RNA-seq transcript/read data is mapped to locations on the genome by alignment where the transcription took place. For finding slopes, we first calculate the cumulative frequency of reads for an operon. We then find slopes-sl1 (that would represent the average read increase per window) for the whole region. RNA-seq data show that even when the read coverage falls drastically in the IR implying a termination has occurred, the reads are not completely absent in the region beyond the putative termination site. Therefore, to identify the termination site, all the regions where the count of reads increase after the stop codon is found. So, if we take the adjacent differences of sl1 slope values, we are taking the second derivative of the cumulative frequency curves, defined by sl2.

### Calculating slope sl1

Let *a* and *b* be two points on cumulative frequency plot at positions (*i*) and (*i* + *w*), where *w* is the varying window size. Let read frequency at *a* be *af* and at *b* be *bf*.

$${\text{Slope }} = \, \left( {y2 - y1} \right) \, / \, \left( {x2 - x1} \right).{\text{ So}},{\text{ here slope would be }}\left( {bf - af} \right)/w$$

We calculated slope values for different windows ‘*w*’ ranging from 5, 10, 15 and so on up to 30. An example case is shown in Figure S14.

### Calculating slope sl2

Let *A* = {*a1*, *a2*, *a3*, *a4*, … *n*} be a set of sl1 slopes for IR region of an operon.

Then sl2 = {*a2*-*a1*, *a3*-*a2*, *a4*-*a3*, … *an*-*a*(*n*-1)).

sl2 would have positive and negative values as well as saddle points (points of inflection). In the IR we find the first negative sl2 slope after the stop codon which is followed by the positive sl2 slope. We take such saddle point and extend it till another drop in slope is seen.

The changes in slope are prominently visible early in the IR using window sizes 5, 10, and sometimes 15, but many such inflection points are lost when higher lengths of the window, i.e. 20–30 are taken. So, we take 10 as the window size for our analysis (Fig. S14).

Now, we rationalize that termination must have taken place at the start of such a region. This is because, the drastic decrease in read frequency points to termination, and in order for an efficient termination, the cell is likely to pick an early termination site, and hence we too choose the first such region for the presence of hairpin termination units. Taking w = 10, if the average of all slope values are calculated in the coding region then it is the coding (operon) region slope and if it is calculated in IR then it is IR slope. IR slope values range from 0 to 400 in our data, while coding region slope values range from 10^4^ to 10^6^. By taking the ratio of IR slope values : coding region slope values; i.e. 0–400: 10^4^–10^6^, would be in the range of ~ 0 to 0.004. showing that even though the slope values are positive in IR, the marginal increase of reads is negligible.

### Segmenting the IRs

The positive slope region (pos) divides the IR into three zones; Zone 1, Zone 2, and Zone 3, marked with the help of IR^st^, pos^st^, pos^end^, and IR^end^, which represent IR start, positive slope region start, positive slope region end, and IR end, respectively (Fig. 1). The *single* and *cluster* units are placed in one of the three zones, based on their start location on the genome and position with respect to the positive slope region. All hairpins that start before pos^st^, fall in Zone 1; those starting between pos^st^ and pos^end^ fall in Zone 2, and those starting after pos^end^ fall in Zone 3. The *cluster* and *single* that are closest to pos^st^ are labeled as *RNA-seq derived* hairpins. These hairpins determine the most likely experimental transcription termination site of the gene/operon. A recent study^84,85^ have similarly used RNA-seq data for inference of ITT site prediction in archea, where it has shown in vivo that reads form valid termination sites with additional T stretch or hairpins present upstream.

### Matching hairpin(s) for termination site confirmation

**Figure 10:**
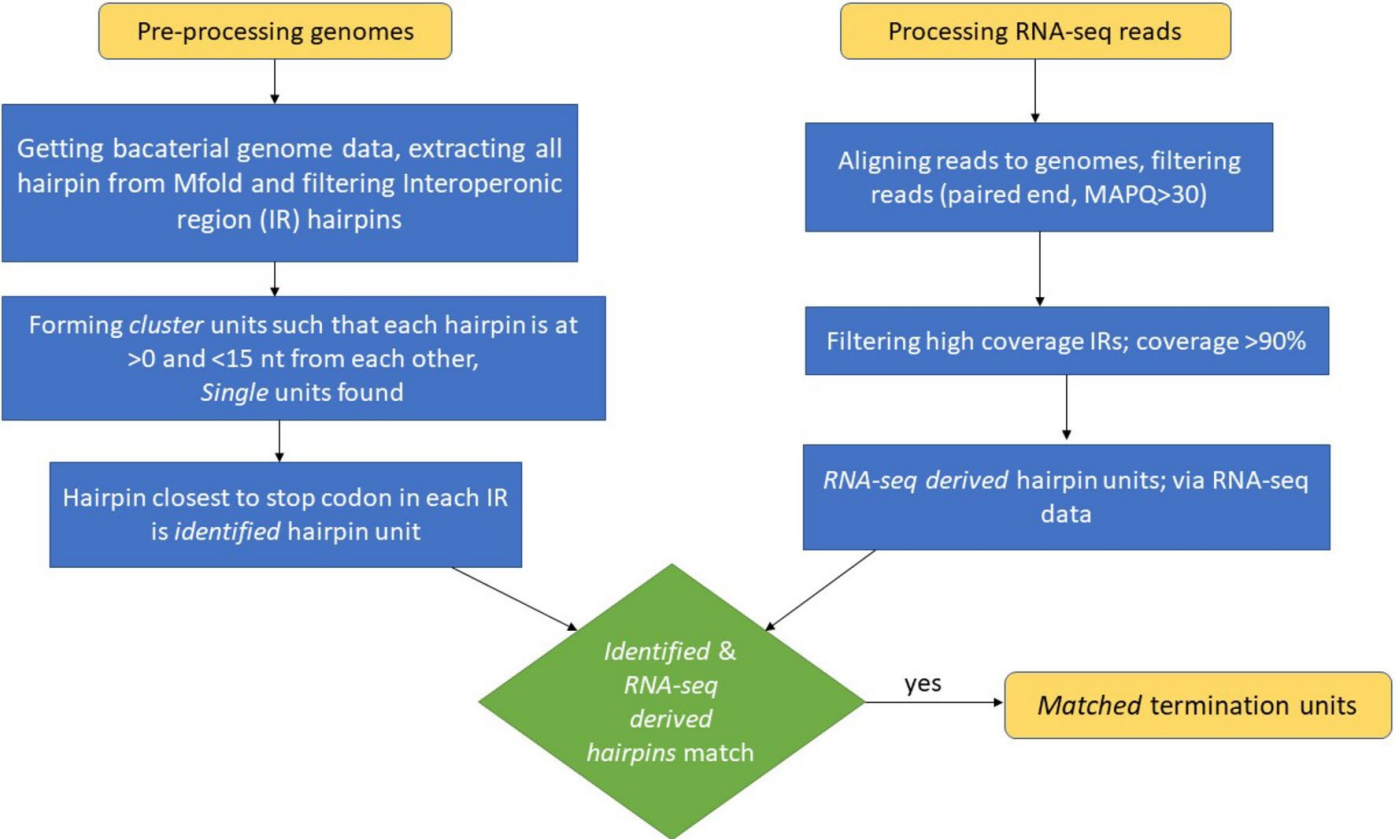
Workflow of our analysis. shows a flowchart to see the steps used to arrive at *matched* termination units and shows how *identified* and *RNA-seq derived* termination units are found.

A hairpin is expected to terminate transcription around the start of the positive slope region. Accordingly, the criterion that the termination must occur within the ± ‘n’ bases of the detected positive slope region start to count for a true prediction is applied. A *cluster* that straddles across Zone 1 and Zone 2 has n = 0, and the one restricted to Zone 1, the n value is calculated from the last base of the last hairpin, while for Zone 2 it is the first base of the first hairpin (similar to as shown in Fig. 4c,d). As the absolute value of n increases, the likelihood of correct site identification decreases. Although few, predictions in Zone 3 are far from the stop codon and mostly erroneous. For these cases, the MFold software is unable to predict a hairpin early in the IR and thus excluded for calculating the accuracy of our method. The workflow for finding *matched* hairpins is shown as a flowchart in Fig. 10.

### Finding poly U/poly A trails

We have taken DNA sequences and replaced Ts with Us. We then check for As and Us to take care of genes both on the sense (+) and anti-sense (−) strands. The poly U or poly A pattern associated with the intrinsic terminators is supposed to be a continuous stretch that is not often interspersed with other bases. Therefore, for pattern search, we use a minimum criterion that at least three consecutive A or U bases must be present to label it as a true site in the IR. The pattern may not be perfect and can have mismatches with the presence of other bases in between. To account for this, we check for patterns with ≤ 1 and ≤ 2 mismatches as well. If there is more than one pattern in the IR, the one nearest to the upstream hairpin is taken. These are further divided into *clusters* located at various distances from the nearest upstream hairpin. We similarly also searched for the presence of poly G/C patterns downstream of the *identified* hairpin units.

### Comparing *Identified* hairpin units with Molquest terminator site prediction

Along with operon prediction, Molquest also gives predictions for ITT sites in bacterial genomes. This feature was used to predict hairpins for the 13 bacterial genomes used in our analysis and the results obtained were compared with hairpins identified by our algorithm. In 44% of IRs both Molquest and our method predicted hairpin sites (common); in 7%, neither could make any prediction, while in 6% and 43% cases Molquest and our method, respectively, made exclusive predictions. For common case predictions, very few sites had a match comparing the distance between midpoints of the predicted terminators (Fig. S15). Here 69% of the IRs had our *identified* termination units located ahead of Molquest predicted terminators. Our exclusive predictions are primarily comprised of *cluster* cases. In 10 out of 13 genomes, > 50% of termination units corresponded to *cluster* with as high as 77% in *P. aeruginosa*.

### Ethics approval and consent to participate

This work is entirely computational and does not require any approval.

### Consent for publications

All authors have read and approved the manuscript for publication.

## Supplementary Information

Supplementary Information 1.

## Data Availability

The work is done from the data available from the public repository. The authors will share any additional data on request.
