# Supplementary material for: Clusters of hairpins induce intrinsic transcription termination in bacteria
Source: Sci Rep. 2021 Aug 10;11:16194. doi: 10.1038/s41598-021-95435-3 (PMC8355165; doi:10.1038/s41598-021-95435-3)
Supplement: Supplementary file 1 — Supplementary Information 1. [file 41598_2021_95435_MOESM1_ESM.pdf]

Supplementary File

Clusters of hairpins induce intrinsic transcription termination in bacteria

Swati Gupta[1] and Debnath Pal[1*]

[1]Department of Computational and Data Sciences, Indian Institute of Science, Bengaluru, Karnataka 560012, India.

* Corresponding author

E-mail: dpal@iisc.ac.in

Supplementary File

Table S1. Classification of transcription termination studies based on the type of methods used and the organism(s) involved

Citation[#]	Method used			Organism
	In vitro	*In vivo*	*In silico*	
(Unniraman, et al., 2001) (GeSTer)	Yes	Yes	Yes	*M. tuberculosis*, *M. smegmatis*
(Ingham, et al., 1995)	Yes	Yes		*Streptomyces coelicolor* A3, *S. lividans* 1326
(Mitra, et al., 2009) (GeSTer)			Yes	Multiple
(Castillo, et al., 2008)		Yes	Yes	*H. pylori*
(Pulido and Jimenez, 1987)		Yes		*S. lividans*
(Deng, et al., 1987)		Yes		*S. lividans*
(Abe and Aiba, 1996)		Yes		*E. coli*
(Reynolds and Chamberlin, 1992)		Yes		*E. coli*
(Roberts, 2019) (review)	Yes			*E. coli*
(Reynolds, et al., 1992)	Yes			*E. coli*
(Yarnell and Roberts, 1999)	Yes	Yes		*E. coli*
(Wang, et al., 2019)	Yes	Yes		*E.coli* gal
(Li, et al., 2016)		Yes		*E. coli*
(Mader, et al., 2004)	Yes			*B. subtilis*
(Zamudio, et al., 2014)		Yes		Mouse
(Reines, et al., 1987)	Yes			human H3.3 cDNA clone, *E. coli*
(Churchman and Weissman, 2011) (NET-seq)		Yes		*S. cerevisiae* RNAPII
(Clarke, et al., 2018) (NET-seq)	Yes	Yes		*S. cerevisiae RNAP*I, seq from *E. coli*
(Serganov and Patel, 2007) (Review)	Yes	Yes		Multiple prokaryotes, eukaryote
(Brantl, 2004)(review)				*B. subtilis*, *E. coli*
(Cheah, et al., 2007)		Yes		fungus *Neurospora crassa*
(Winkler, et al., 2004)		Yes		*B. subtilis*
(Winkler and Breaker, 2003)	Yes	Yes		*B. subtilis*, *E. coli*
(Henkin, 2000) (Review)				Multiple
(McDaniel, et al., 2003)	Yes	Yes		*B. subtilis*
(de Hoon, et al., 2005)			Yes	*B. subtilis* and 56 other firmicutes
(Washburn, et al., 2001)		Yes		*S. aureus*
(Czyz, et al., 2014)	Yes			*M. bovis*
(Kingsford, et al., 2007) (Transterm hp)			Yes	Multiple
(Adhya and Gottesman, 1978) (Review)				
(Ermolaeva, et al., 2000) (Transterm)			Yes	Multiple
(Besemer, et al., 2001)			Yes	Multiple
(Ciampi, 2006) (Review)	Yes			Multiple
(Quirk, et al., 1993)		Yes		*B. subtilis*
(Mitra, et al., 2017) (Review)	Yes	Yes		*E. coli*, *Salmonella enterica*

# References

Abe, H. and Aiba, H. Differential contributions of two elements of rho-independent terminator to transcription termination and mRNA stabilization. *Biochimie* 1996;78(11-12):1035-1042.
Adhya, S. and Gottesman, M. Control of transcription termination. *Annu Rev Biochem* 1978;47:967-996.
Besemer, J., Lomsadze, A. and Borodovsky, M. GeneMarkS: a self-training method for prediction of gene starts in microbial genomes. Implications for finding sequence motifs in regulatory regions. *Nucleic Acids Res* 2001;29(12):2607-2618.
Brantl, S. Bacterial gene regulation: from transcription attenuation to riboswitches and ribozymes. *Trends Microbiol* 2004;12(11):473-475.
Castillo, A.R.*, et al.* Experimental analysis of Helicobacter pylori transcriptional terminators suggests this microbe uses both intrinsic and factor-dependent termination. *Mol Microbiol* 2008;67(1):155-170.

Supplementary File

Cheah, M.T., *et al.* Control of alternative RNA splicing and gene expression by eukaryotic riboswitches. *Nature* 2007;447(7143):497-500.
Churchman, L.S. and Weissman, J.S. Nascent transcript sequencing visualizes transcription at nucleotide resolution. *Nature* 2011;469(7330):368-373.
Ciampi, M.S. Rho-dependent terminators and transcription termination. *Microbiology* 2006;152(Pt 9):2515-2528.
Ciccarelli, F.D., *et al.* Toward automatic reconstruction of a highly resolved tree of life. *science* 2006;311(5765):1283-1287.
Clarke, A.M., *et al.* NETSeq reveals heterogeneous nucleotide incorporation by RNA polymerase I. *Proc Natl Acad Sci U S A* 2018;115(50):E11633-E11641.
Czyz, A., *et al.* Mycobacterial RNA polymerase requires a U-tract at intrinsic terminators and is aided by NusG at suboptimal terminators. *mBio* 2014;5(2):e00931.
de Hoon, M.J., *et al.* Prediction of transcriptional terminators in Bacillus subtilis and related species. *PLoS Comput Biol* 2005;1(3):e25.
Deng, Z.X., Kieser, T. and Hopwood, D.A. Activity of a Streptomyces transcriptional terminator in Escherichia coli. *Nucleic Acids Res* 1987;15(6):2665-2675.
Ermolaeva, M.D., *et al.* Prediction of transcription terminators in bacterial genomes. *J Mol Biol* 2000;301(1):27-33.
Henkin, T.M. Transcription termination control in bacteria. *Curr Opin Microbiol* 2000;3(2):149-153.
Ingham, C.J., Hunter, I.S. and Smith, M.C. Rho-independent terminators without 3' poly-U tails from the early region of actinophage oC31. *Nucleic Acids Res* 1995;23(3):370-376.
Kingsford, C.L., Ayanbule, K. and Salzberg, S.L. Rapid, accurate, computational discovery of Rho-independent transcription terminators illuminates their relationship to DNA uptake. *Genome Biol* 2007;8(2):R22.
Li, R., *et al.* Effects of cooperation between translating ribosome and RNA polymerase on termination efficiency of the Rho-independent terminator. *Nucleic Acids Res* 2016;44(6):2554-2563.
Mader, U., *et al.* Transcriptional organization and posttranscriptional regulation of the Bacillus subtilis branched-chain amino acid biosynthesis genes. *J Bacteriol* 2004;186(8):2240-2252.
McDaniel, B.A., *et al.* Transcription termination control of the S box system: direct measurement of S-adenosylmethionine by the leader RNA. *Proc Natl Acad Sci U S A* 2003;100(6):3083-3088.
Mitra, A., *et al.* Occurrence, divergence and evolution of intrinsic terminators across eubacteria. *Genomics* 2009;94(2):110-116.
Mitra, P., *et al.* Rho Protein: Roles and Mechanisms. *Annu Rev Microbiol* 2017;71:687-709.
Pulido, D. and Jimenez, A. Optimization of gene expression in Streptomyces lividans by a transcription terminator. *Nucleic Acids Res* 1987;15(10):4227-4240.
Quirk, P.G., *et al.* Identification of a putative Bacillus subtilis rho gene. *J Bacteriol* 1993;175(24):8053.
Reines, D., *et al.* Identification of intrinsic termination sites in vitro for RNA polymerase II within eukaryotic gene sequences. *Journal of molecular biology* 1987;196(2):299-312.
Reynolds, R., Bermudez-Cruz, R.M. and Chamberlin, M.J. Parameters affecting transcription termination by Escherichia coli RNA polymerase. I. Analysis of 13 rho-independent terminators. *J Mol Biol* 1992;224(1):31-51.
Reynolds, R. and Chamberlin, M.J. Parameters affecting transcription termination by Escherichia coli RNA. II. Construction and analysis of hybrid terminators. *J Mol Biol* 1992;224(1):53-63.
Roberts, J.W. Mechanisms of Bacterial Transcription Termination. *J Mol Biol* 2019;431(20):4030-4039.
Serganov, A. and Patel, D.J. Ribozymes, riboswitches and beyond: regulation of gene expression without proteins. *Nat Rev Genet* 2007;8(10):776-790.
Unniraman, S., Prakash, R. and Nagaraja, V. Alternate paradigm for intrinsic transcription termination in eubacteria. *J Biol Chem* 2001;276(45):41850-41855.
Wang, X., *et al.* Processing generates 3' ends of RNA masking transcription termination events in prokaryotes. *Proc Natl Acad Sci U S A* 2019;116(10):4440-4445.
Washburn, R.S., *et al.* rho is not essential for viability or virulence in Staphylococcus aureus. *Antimicrob Agents Chemother* 2001;45(4):1099-1103.
Winkler, W.C. and Breaker, R.R. Genetic control by metabolite-binding riboswitches. *Chembiochem* 2003;4(10):1024-1032.

Supplementary File

Winkler, W.C., *et al.* Control of gene expression by a natural metabolite-responsive ribozyme. *Nature* 2004;428(6980):281-286.

Yarnell, W.S. and Roberts, J.W. Mechanism of intrinsic transcription termination and antitermination. *Science* 1999;284(5414):611-615.

Zamudio, J.R., Kelly, T.J. and Sharp, P.A. Argonaute-bound small RNAs from promoter-proximal RNA polymerase II. *Cell* 2014;156(5):920-934.

Supplementary File

Table S2. Change in the number of IRs at different levels of filtering

Organism	Forward strand			Reverse strand		
	**Total IRs**	**Read coverage of coding >90%**	**After outlier removal***	**Total IRs**	**Read coverage of coding >90%**	**After outlier removal***
Staphylococcus aureus Newman	759	481	441	763	471	429
Clostridium phytofermentans ISDg	1350	719	662	1186	651	597
Mycobacterium gilvum PYR-GCK	1372	702	648	1425	716	660
Bifidobacterium pseudocatenulatum DSM 20438	618	272	250	576	237	218
Treponema denticola ATCC 35405	617	293	265	710	378	347
Leptospira interrogans Manilae L495	1197	700	644	1225	760	694
Fusobacterium nucleatum ATCC 25586	447	240	219	438	260	236
Nostoc punctiforme PCC 73102	2482	1327	1222	2583	1342	1242
Prochlorococcus marinus MIT 9313	898	466	431	807	408	372
Nodularia spumigena UHCC 0039	1676	957	884	1758	1041	959
Pseudomonas aeruginosa PAO1	1604	841	767	1655	890	818
Syntrophus aciditrophicus SB	959	532	492	898	500	463
Klebsiella pneumoniae UHKPC07	1575	426	392	1500	417	386

*outlier IRs which lie in the 5% tail region of the average read depth histogram of CDS or IR regions.

Supplementary File

Table S3. Statistics on the occurrence of genes and operons in the bacterial genomes studied

Organism	#genes	#operons	Average genes per operon
Staphylococcus aureus Newman	2613	1522	1.7
Clostridium phytofermentans ISDg	3901	2536	1.5
Mycobacterium gilvum PYR-GCK	5240	2797	1.9
Bifidobacterium pseudocatenulatum DSM 20438	1758	1194	1.5
Treponema denticola ATCC 35405	2766	1327	2.1
Leptospira interrogans Manilae L495	3776	2422	1.6
Fusobacterium nucleatum ATCC 25586	2061	885	2.3
Nostoc punctiforme PCC 73102	6051	5065	1.2
Prochlorococcus marinus MIT 9313	2267	1705	1.3
Nodularia spumigena UHCC 0039	4410	3434	1.3
Pseudomonas aeruginosa PAO1	5569	3259	1.7
Syntrophus aciditrophicus SB	3165	1857	1.7
Klebsiella pneumoniae UHKPC07	5142	3075	1.7

Supplementary File

Table S4. Distribution of the lengths of each Zone in the IR for all the genomes in our data set

Zone Category	Distance from the stop codon (bp)							
**Zone 1**	**0-20**	**20-40**	**40-70**	**70-100**	**100-150**	**150-200**	**>200**	
Staphylococcus aureus Newman	121	256	274	127	65	11	5	
Clostridium phytofermentans ISDg	84	231	254	192	191	114	135	
Mycobacterium gilvum PYR-GCK	178	381	361	180	107	29	31	
Bifidobacterium pseudocatenulatum	55	120	128	81	35	10	5	
Treponema denticola ATCC 35405	85	186	160	77	42	14	7	
Leptospira interrogans Manilae L495	213	371	411	192	86	23	13	
Fusobacterium nucleatum ATCC	54	119	102	80	47	14	13	
Nostoc punctiforme PCC 73102	376	701	661	346	243	54	38	
Prochlorococcus marinus MIT 9313	166	229	286	82	26	3	1	
Nodularia spumigena UHCC 0039	285	561	530	271	111	23	25	
Pseudomonas aeruginosa PAO1	193	443	471	223	173	34	10	
Syntrophus aciditrophicus SB	168	302	257	122	53	7	8	
Klebsiella pneumoniae UHKPC07	87	203	167	99	79	37	40	
**Zone 2**	**0-20**	**20-40**	**40-70**	**70-100**	**100-150**	**150-200**	**>200**	
Staphylococcus aureus Newman	312	313	149	54	31	0	0	
Clostridium phytofermentans ISDg	389	542	165	52	37	15	2	
Mycobacterium gilvum PYR-GCK	489	447	202	62	63	4	0	
Bifidobacterium pseudocatenulatum	144	112	56	78	42	2	1	
Treponema denticola ATCC 35405	205	218	88	29	29	0	2	
Leptospira interrogans Manilae L495	563	453	180	91	20	1	1	
Fusobacterium nucleatum ATCC	131	181	71	20	25	2	0	
Nostoc punctiforme PCC 73102	715	800	675	192	36	1	0	
Prochlorococcus marinus MIT 9313	295	365	123	10	0	0	0	
Nodularia spumigena UHCC 0039	679	750	244	56	71	6	0	
Pseudomonas aeruginosa PAO1	703	485	208	56	87	6	2	
Syntrophus aciditrophicus SB	381	276	198	48	13	1	0	
Klebsiella pneumoniae UHKPC07	229	328	107	45	5	0	0	
**Zone 3**	**0-20**	**20-40**	**40-70**	**70-100**	**100-**	**200-**	**400-**	**800-**
Staphylococcus aureus Newman	47	16	35	60	170	198	91	158
Clostridium phytofermentans ISDg	108	30	86	72	229	219	97	209
Mycobacterium gilvum PYR-GCK	59	36	59	57	98	101	135	469
Bifidobacterium pseudocatenulatum	46	15	27	27	63	50	40	101
Treponema denticola ATCC 35405	68	19	47	41	78	47	34	128
Leptospira interrogans Manilae	47	23	51	56	101	183	232	392
Fusobacterium nucleatum ATCC	106	16	39	38	60	23	27	67
Nostoc punctiforme PCC 73102	138	51	134	125	307	368	314	614
Prochlorococcus marinus MIT 9313	22	12	38	36	88	118	110	259
Nodularia spumigena UHCC 0039	82	51	105	129	310	272	182	406
Pseudomonas aeruginosa PAO1	165	46	102	104	185	125	117	424
Syntrophus aciditrophicus SB	56	30	59	71	159	134	86	187
Klebsiella pneumoniae UHKPC07	81	14	26	23	56	46	60	253

Supplementary File

Table S5. Distribution of *identified* terminations units downstream of the stop codon expressed as a cumulative percentage

**Termination unit**	**Distance from stop codon (bp)**					
***Cluster* hairpin**	**0 - 20**	**20 - 50**	**50 - 80**	**80 - 110**	**110 - 150**	**>150**
S. aureus Newman	57.95	88.64	96.97	98.48	98.86	100.00
C. phytofermentans ISDg	61.97	88.24	95.59	97.27	97.90	100.00
M. gilvum PYR-GCK	83.18	95.11	98.75	99.43	99.55	100.00
B. pseudocatenulatum DSM 20438	77.10	94.39	97.20	98.13	98.60	100.00
T. denticola ATCC 35405	61.73	87.36	97.47	99.28	99.64	100.00
L. interrogans Manilae L495	62.50	91.62	96.95	98.93	99.24	100.00
F. nucleatum ATCC 25586	45.70	71.52	92.72	98.01	99.34	100.00
N. punctiforme PCC 73102	64.84	90.04	97.22	99.19	99.82	100.00
P. marinus MIT 9313	71.06	92.44	97.84	98.27	98.49	100.00
N. spumigena UHCC 0039	60.16	88.58	96.69	99.09	99.20	100.00
P. aeruginosa PAO1	77.19	93.16	99.33	99.52	99.52	100.00
S. aciditrophicus SB	75.05	94.70	98.43	99.21	99.41	100.00
K. pneumoniae UHKPC07	61.82	85.47	97.40	99.57	99.78	100.00
Mean	66.17	89.33	97.12	98.80	99.18	100.00
Standard Deviation	10.11	6.16	1.63	0.70	0.57	0.00
***Single* hairpin**	**0 - 20**	**20 - 50**	**50 - 80**	**80 - 110**	**110 - 150**	**>150**
S. aureus Newman	38.90	89.89	98.68	99.56	99.78	100.00
C. phytofermentans ISDg	49.83	90.33	97.50	99.17	99.67	100.00
M. gilvum PYR-GCK	68.40	96.74	98.37	99.35	99.35	100.00
B. pseudocatenulatum DSM 20438	66.49	94.59	99.46	99.46	99.46	100.00
T. denticola ATCC 35405	50.88	92.92	98.23	99.56	99.56	100.00
L. interrogans Manilae L495	62.25	92.29	97.23	99.21	99.60	100.00
F. nucleatum ATCC 25586	39.13	87.50	95.65	100.00	100.00	100.00
N. punctiforme PCC 73102	57.98	90.98	97.92	99.11	99.21	100.00
P. marinus MIT 9313	65.77	92.79	96.85	98.20	98.65	100.00
N. spumigena UHCC 0039	56.91	88.32	96.64	98.93	99.33	100.00
P. aeruginosa PAO1	71.83	93.28	98.45	99.22	99.22	100.00
S. aciditrophicus SB	64.82	91.53	98.05	99.35	99.35	100.00
K. pneumoniae UHKPC07	65.00	93.85	98.85	99.23	99.23	100.00
Mean	58.32	91.93	97.84	99.26	99.42	100.00
Standard deviation	10.74	2.55	1.04	0.41	0.33	0.00

Supplementary File

Table S6. Statistics of *RNA-seq derived* and predicted hairpins that match when we apply a missed termination criterion for the *identified* hairpin site

Organism	#*RNA-seq derived*	#Match with predicted
S. aureus Newman	671	654
C. phytofermentans ISDg	991	955
M. gilvum PYR-GCK	1135	1089
B. pseudocatenulatum DSM 20438	364	360
T. denticola ATCC 35405	455	441
L. interrogans Manilae L495	1090	1064
F. nucleatum ATCC 25586	293	288
N. punctiforme PCC 73102	1996	1948
P. marinus MIT 9313	646	633
N. spumigena UHCC 0039	1491	1456
P. aeruginosa PAO1	1378	1332
S. aciditrophicus SB	773	756
K. pneumoniae UHKPC07	631	611
Total	11914	11587

Supplementary File

Table S7. Statistics showing the cumulative percentage of operons with poly U/A pattern (with at least 3 consecutive identical nucleotides) at a given distance from the hairpin end

Organism	Distance from the hairpin end (bp)								
	[0-2)	[2-4)	[4-6)	[6-8)	[8-10)	[10-15)	[15-20)	[20-30)	>30
S. aureus Newman	5.19	15.70	24.97	33.38	42.51	57.29	65.18	73.59	81.21
C. phytofermentans ISDg	4.61	15.06	23.46	31.15	39.27	53.86	62.22	72.28	81.59
M. gilvum PYR-GCK	0.64	2.11	3.54	4.86	6.29	10.26	12.58	18.41	65.46
B. pseudocatenulatum DSM 20438	1.76	7.79	12.73	17.00	23.53	33.84	40.62	49.41	78.98
T. denticola ATCC 35405	5.28	17.78	26.75	34.74	42.65	56.74	64.13	72.12	82.82
L. interrogans Manilae L495	6.65	22.42	33.20	43.15	51.03	66.02	73.95	80.64	85.59
F. nucleatum ATCC 25586	5.54	17.85	28.59	38.76	45.42	58.08	63.84	68.59	72.54
N. punctiforme PCC 73102	4.34	13.35	21.03	26.50	33.17	47.17	55.18	64.90	81.90
P. marinus MIT 9313	2.82	8.68	13.37	18.06	23.28	33.55	41.06	52.32	83.05
N. spumigena UHCC 0039	4.51	13.83	21.00	26.50	33.34	46.45	54.98	66.72	85.70
P. aeruginosa PAO1	1.38	4.79	7.24	8.93	11.81	16.97	20.77	28.66	79.04
S. aciditrophicus SB	3.66	11.52	18.36	24.50	32.20	43.35	53.10	64.40	84.71
K. pneumoniae UHKPC07	2.93	9.14	14.89	18.89	23.25	33.14	40.49	52.55	88.52
Average	3.79	12.31	19.16	25.11	31.37	42.82	49.85	58.81	80.85

Supplementary File

Table S8. Statistics of orthologous operons found for different bacterial subspecies

S. No.	Bacterial species	Total subspecies	Total operons in all subspecies	Complete operons on same strand	% Orthologous Operons
A	Fusobacteria	13	1632	664	40.69
B	Pseudobacteria	35	16728	4406	26.34
C	Staphylococcus	23	14454	6417	44.4
D	Prochlorococcus	5	752	632	84.04
E	Clostridium	25	8136	3239	39.81
F	Nostoc	4	1287	870	67.6
G	Bifidobacterium	21	6540	2319	35.46
H	Klebsiella	14	2444	661	27.05
I	Mycobacterium	25	2136	677	31.69
J	Treponema	12	1584	584	36.87

More details in Section R1 below.

Supplementary File

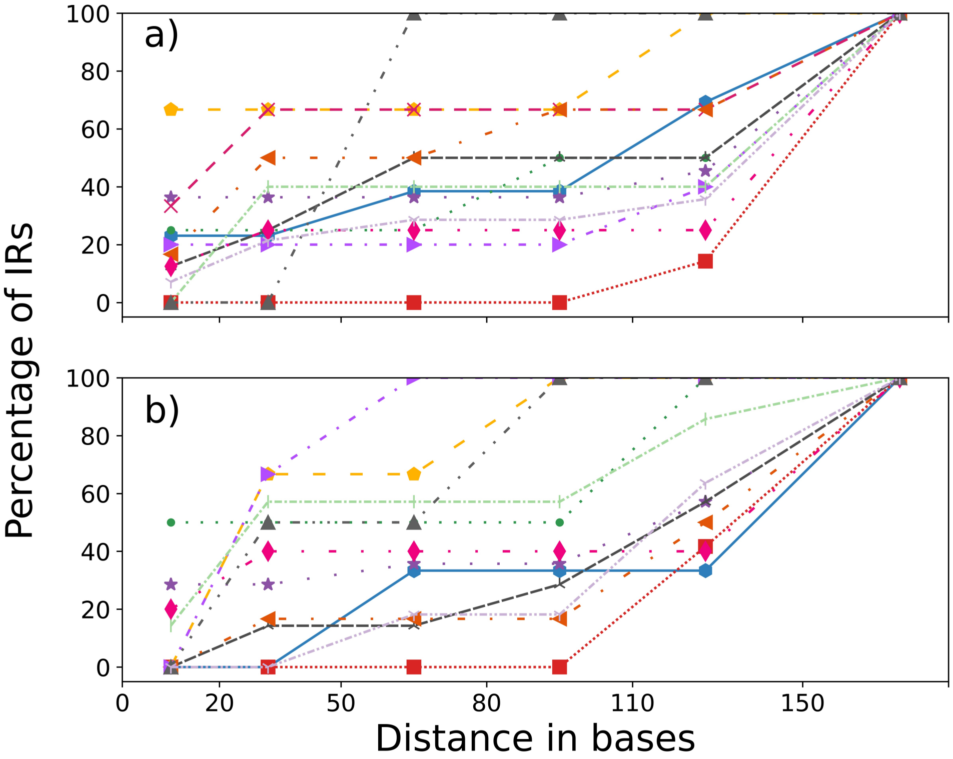

Fig. S1. **Hairpin prediction for rRNA genes.** Line plot showing the distance from the stop codon to *identified* termination units in rRNA genes. The percentage is calculated with respect to total **a)** *cluster* units and in **b)** *single* units, respectively. On average, we found 54% of rRNA genes with *cluster* hairpin and the rest with a *single* hairpin. We found a termination unit for all rRNA genes in 13 bacterial genomes extracted from the Genbank annotated files. Unlike mRNA genes, we see that the hairpins are further away from the stop codon. The total number of genes in the genome is small, a separate study with more genomes is warranted for any statistically significant assessment.

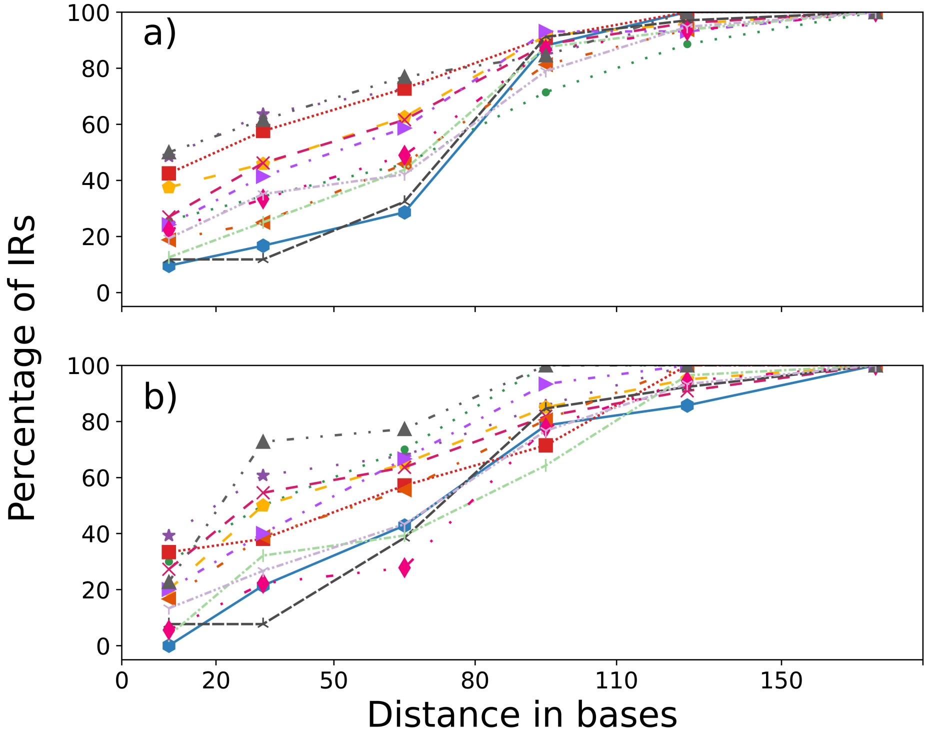

Fig. S2. **Hairpin prediction for tRNA genes.** Line plot showing the distance from the stop codon to *identified* termination units in tRNA genes. The percentage is calculated with respect to total **a)** *cluster* units and in **b)** *single* units, respectively. On average, we found 63% of tRNA genes with *cluster* hairpin and the rest with a *single* hairpin. We could not find a termination unit in one tRNA gene in *M. gilvum*. As with rRNA, here too we find hairpins located further downstream of the stop codon. However, the rise in the distance with reference to hairpin frequency is more uniform than in rRNA. The total number of genes in the genome is small, a separate study with more genomes is warranted for any statistically significant assessment.

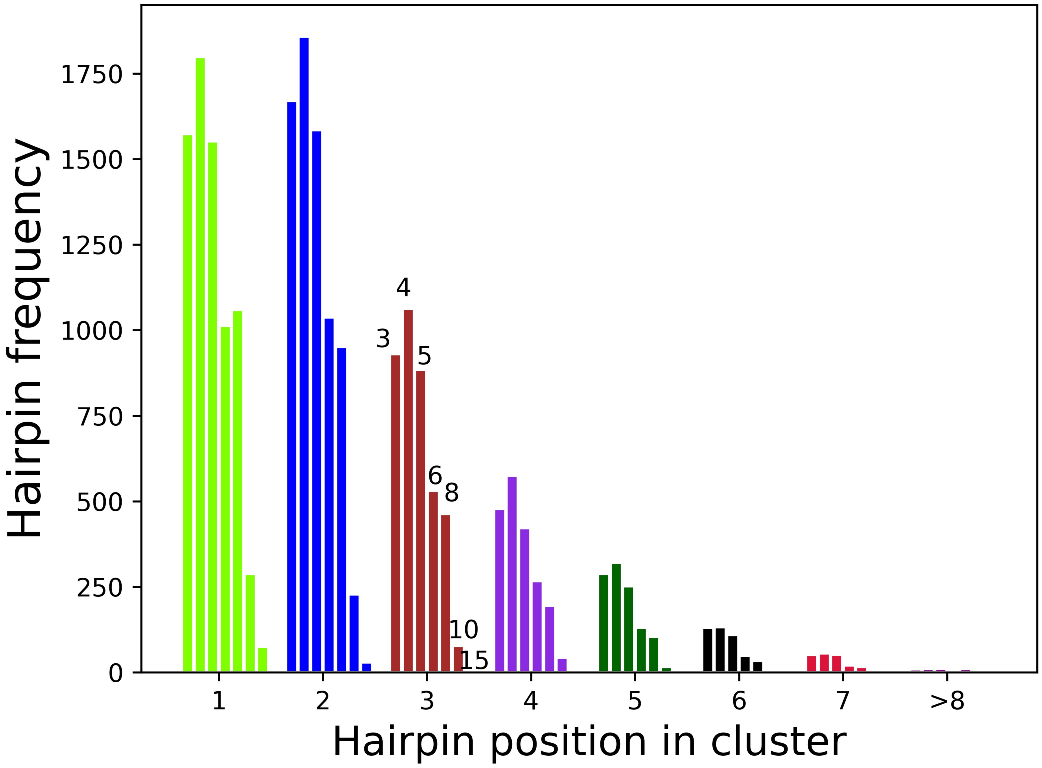

Fig. S3. Distribution of stem lengths with respect to the position of hairpins in a *cluster*. Each hairpin position (x-axis) contains stem lengths for all hairpins at that position in each *cluster* across the 13 genomes analyzed. Each position is a histogram representing stem lengths: 3, 3-4, 4-5, 5-6, 6-8, 8-10, 10-15. For clarity, position 3 bars are marked with upper limit stem length labels. We do not know if the first hairpin alone may be capable of transcription termination or not. This would depend on the strength of the hairpin, and we have seen in some instances where the first hairpin is stronger than the later ones. A short hairpin alone will cause weak termination that may result in a read through unlike in a *cluster*.

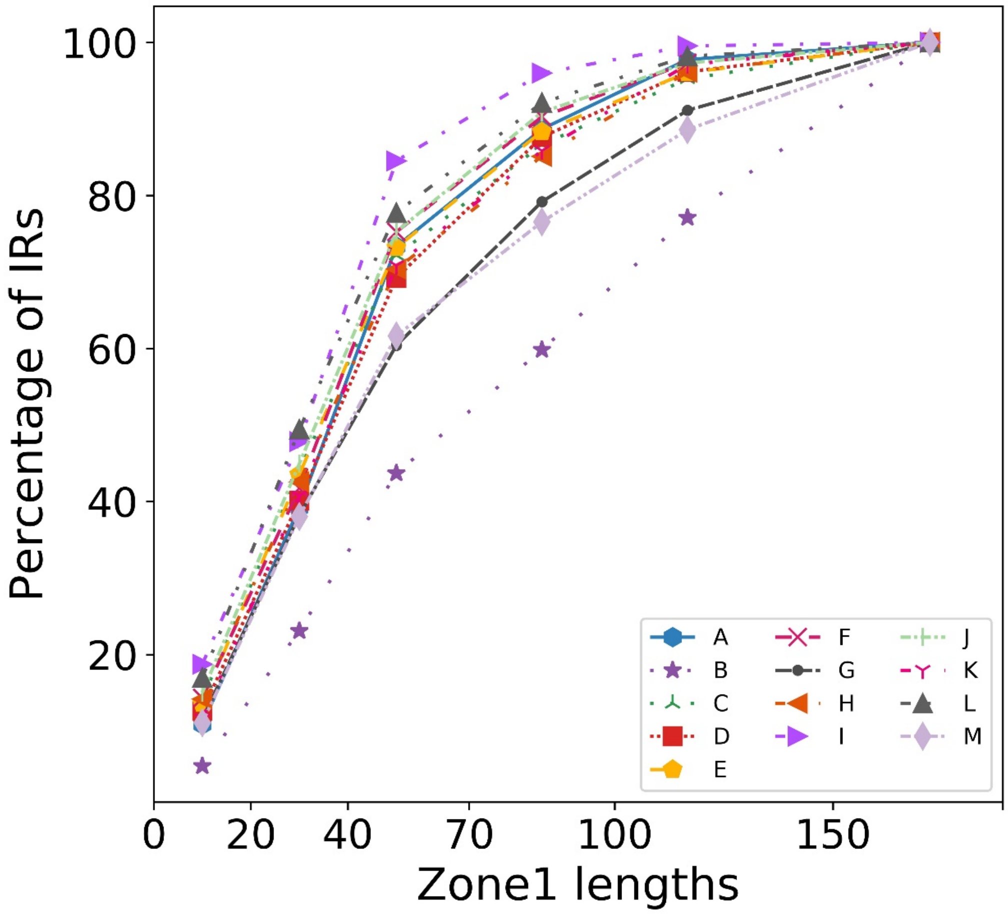

Fig. S4. Zone 1 lengths. Line plots showing the cumulative percentage of IRs for different Zone 1 lengths (in nucleotides) for each genome. The percentages have been calculated with respect to IRs where a positive slope region was found. The values corresponding to each organism from A-M are as follows: 671, 991, 1135, 364, 455, 1090, 293, 1196, 646, 1491, 1378, 773, 631. It can be seen that Zone 1 lengths are quite small, with around 80% IRs having Zone 1 length up to 70 bases.

Supplementary File

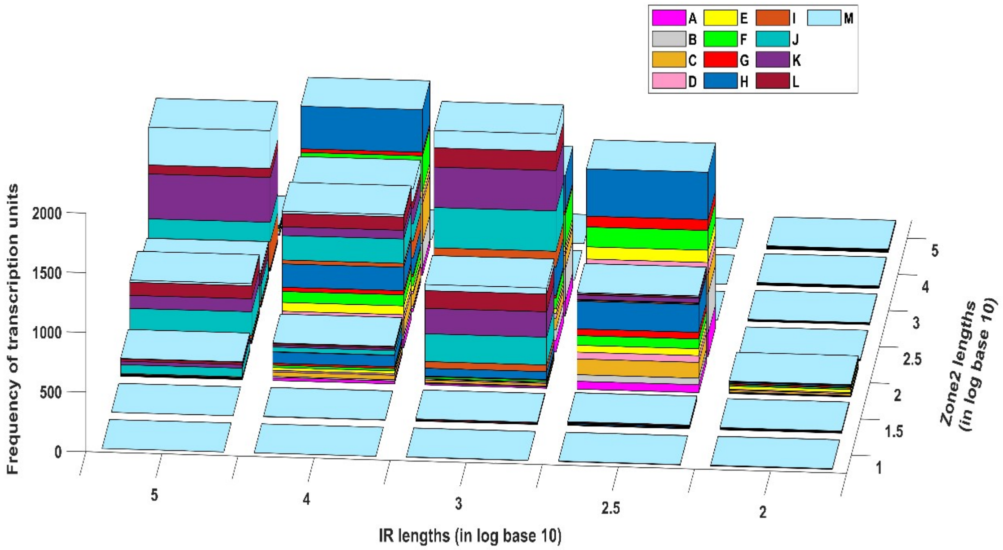

Fig. S5. Zone 2 vs. IR lengths. A 2D histogram showing the relationship and trends between Zone 2 lengths and their respective IR lengths (in log scale) for all *RNA-seq derived* termination units (except those in Zone 3). The different colors represent different genomes as per Table 1.

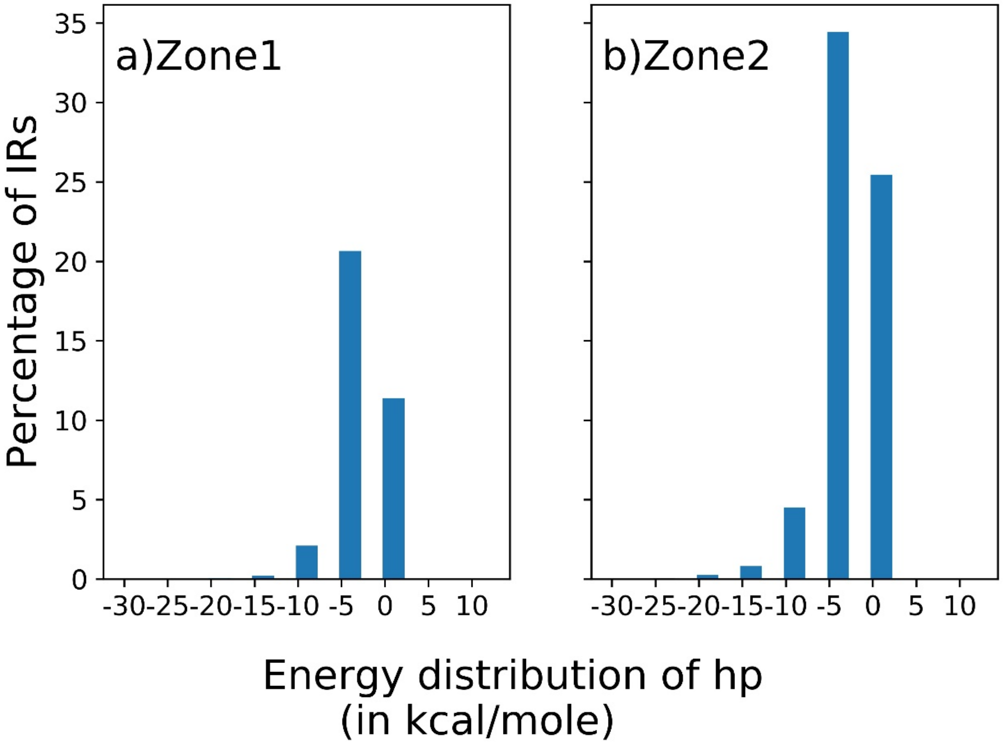

Fig. S6. Energy scores of *RNA-seq derived* hairpin units. Bar graph showing the energy score distribution of all *RNA-seq derived* hairpin units (except those in Zone 3) with **a)** and **b)** showing hairpins in Zone 1 and Zone 2, respectively. The plots have both *single* and *cluster* data where the energy of *cluster* has been found by taking an average of energies of individual hairpins in it. The percentage on the y-axis is calculated with respect to filtered IRs. The score is an estimate of RNA secondary structure stability calculated by the MFold program.

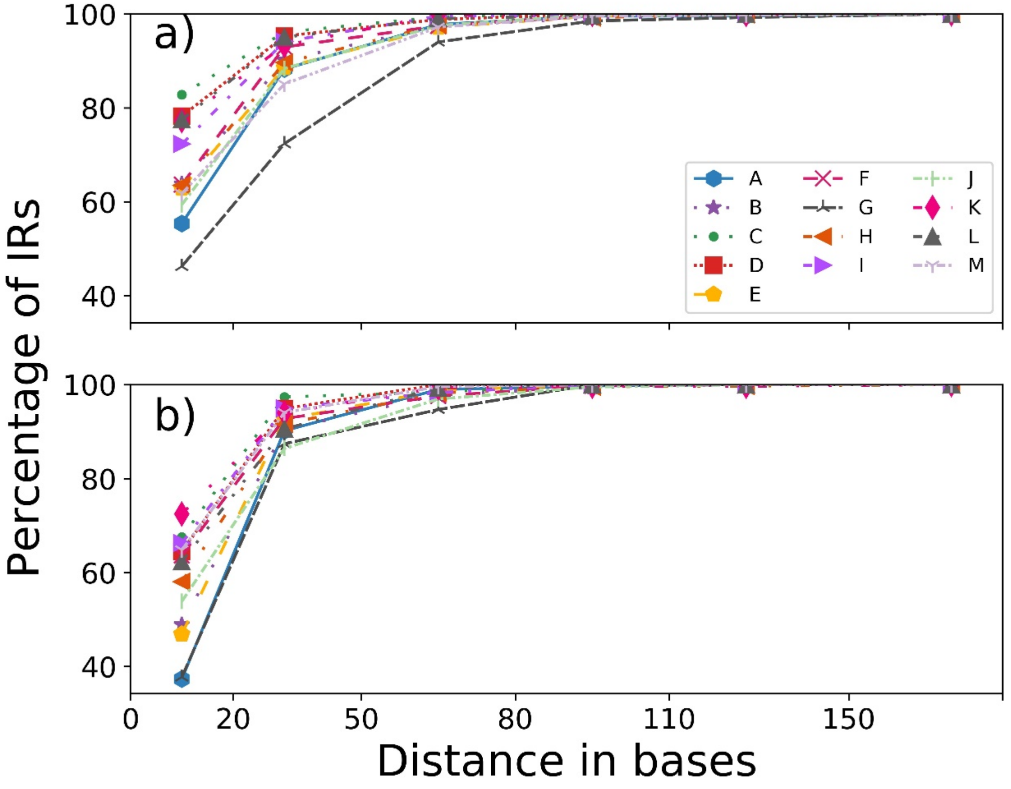

Fig. S7. The distance of hairpins from stop codon. Line plot showing the distance from the stop codon to *matched identified* and *RNA-seq derived* termination units. The percentage is calculated with respect to total **a)** *cluster* units and in **b)** *single* units, respectively.

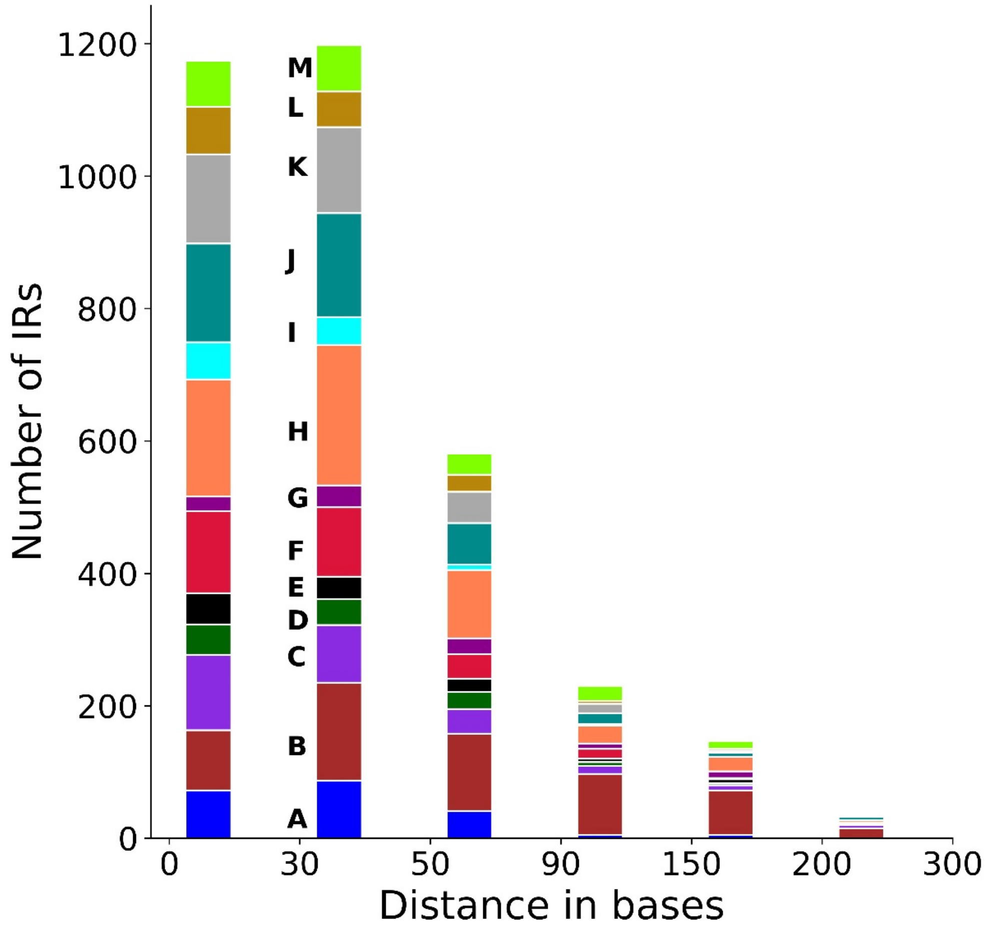

Fig. S8. Distance between *identified* and *RNA-seq derived* hairpin units. Bar graph showing the distance between *identified* and *RNA-seq derived* hairpin units in IRs where both exist but lie at different positions. The different colors represent different bacterial genomes that have been labeled and ordered alphabetically, as in Table 1. Taking the shortest distance between hairpin units to calculate separation distance ensures uniformity in criterion as *cluster* exists in unequal sizes.

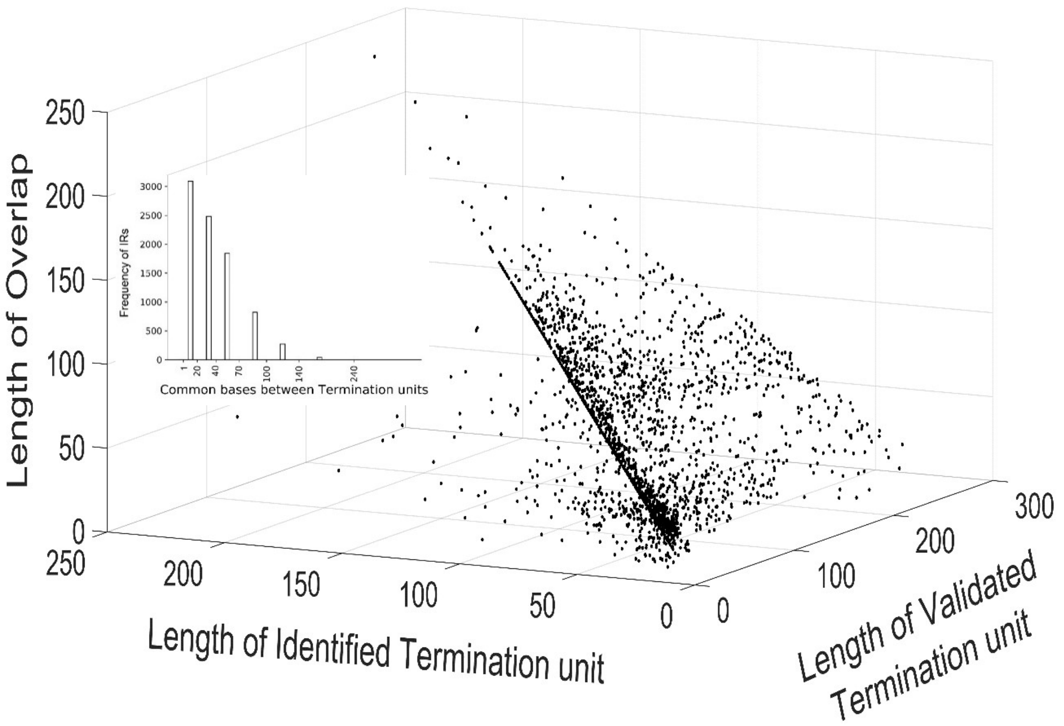

Fig. S9. *Matched* termination units. Scatter plot for *matched* termination units showing the distribution of points corresponding to the length of the *identified* and *RNA-seq derived* termination unit (termed as Validated here), and their overlap length. A majority of the data points are along the diagonal indicating a perfect match. Some termination units are large, which leads to large overlaps as can be seen from the scatter plot. The inset shows a bar graph indicating the frequency of overlap size between the *matched* termination units. The z-axis label of the main plot is common to the base axis label of the inset.

Supplementary File

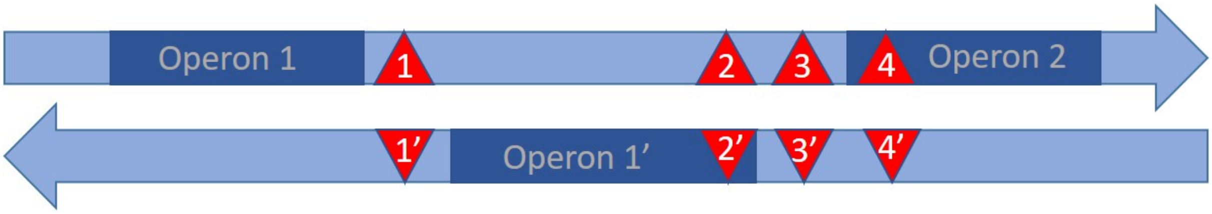

1. **Figure S10. An example arrangement of experimental site terminators.** Example cases of experimental transcription termination zones mapped to the strands from Carafa et al. Schematic diagram showing three operons, 1 and 2 on the forward strand and 1’ in on the reverse strand in a genome. The grey-blue regions are the IRs. The red triangles represent inverted repeat regions/hairpins labeled 1, 2’, 3’, and 4 corresponding to the experimental hairpins. Case 1. The experimental hairpin is correctly located after the stop codon of operon 1. Case 2. Hairpin 2’ location is in the reverse strand operon1’. Case 3. Hairpin 3’ is located before reverse strand operon1’ and next forward strand operon2. Case 4. Hairpin 4 is located in forward strand operon2. The counts of mapped experimental terminator sites from Carafa et al. show a large number of the cases are located close to the end of the IR region as defined by the two operons. which places them in Zone 3 as per our analysis methodology. As a result, they may be part of the 5’ UTR regions of the next operon suggesting that these may be in reality be a pause site.

Reference

d'Aubenton Carafa, Y., Brody, E. & Thermes, C. Prediction of rho-independent Escherichia coli transcription terminators. A statistical analysis of their RNA stem-loop structures. *J Mol Biol* **216**, 835-858 (1990).

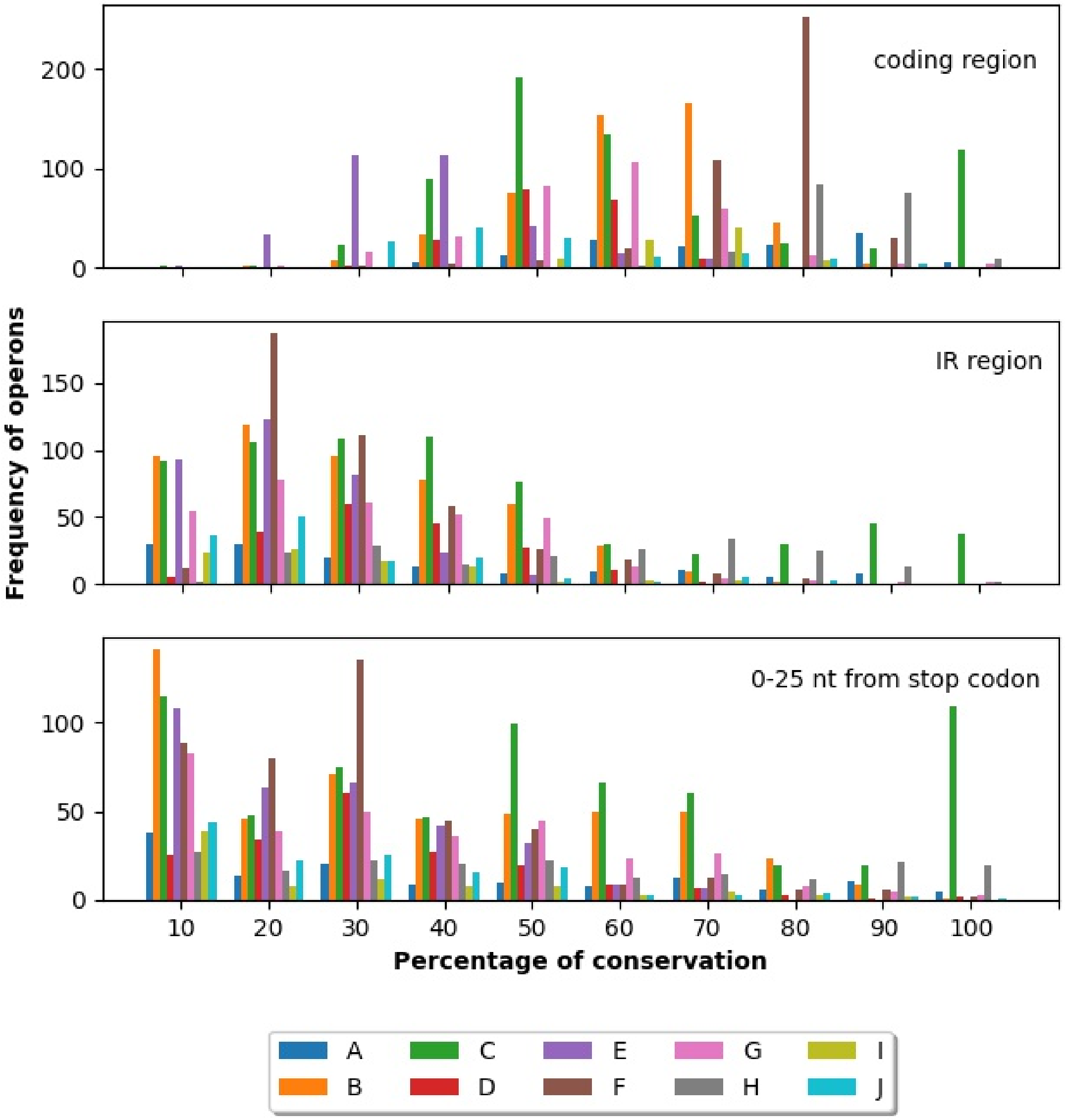

Fig. S11. Conservation of sequence in coding and IR region. Bar plot showing the percentage of conservation in coding and IR region and 0-25 nucleotide segment from the stop codon. The species A-J are as shown in Table S8 above. More details in Section R1 below.

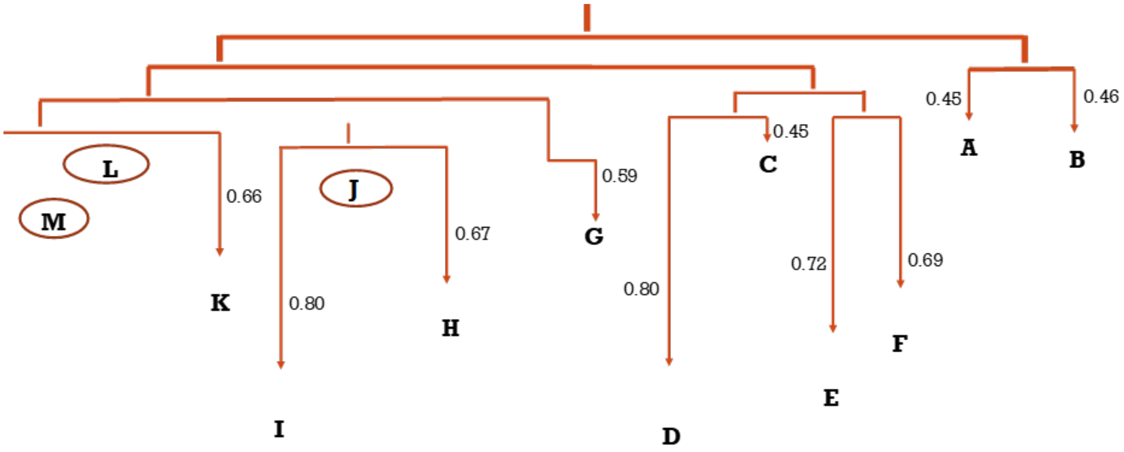

Fig. S12. Phylogenetic tree of the analyzed organisms listed in Table 1 main text. The phylogenetic distance data is obtained from Ciccarelli et al. (Ciccarelli, et al., 2006) and indicated beside the arrows; three genomes shown in oval, have no data in Ciccarelli et al. and are placed in their phyla. At the right of the tree are Firmicutes, the earliest ancestors of all bacteria.

Reference

Ciccarelli FD, Doerks T, Von Mering C, Creevey CJ, Snel B, Bork P. Toward automatic reconstruction of a highly resolved tree of life. science. 2006;311(5765):1283-7.

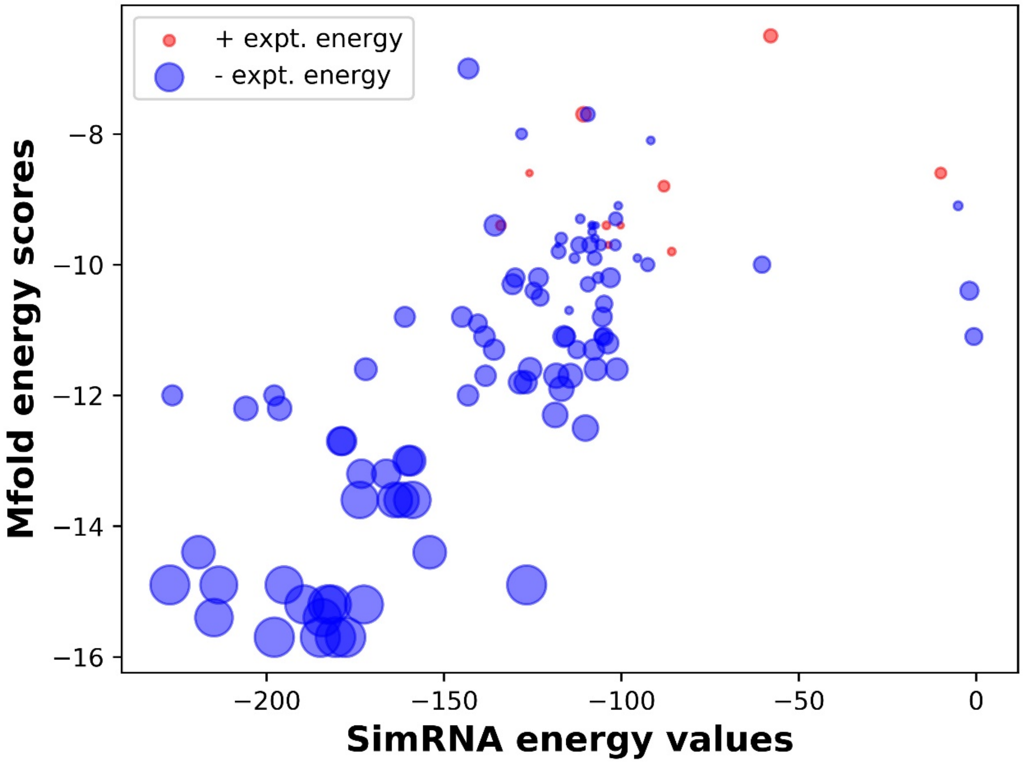

Fig. S13. MFold vs. SimRNA and experimental energy values for experimentally determined hairpins. The size of the red and blue circles signifies the experimental energy values.

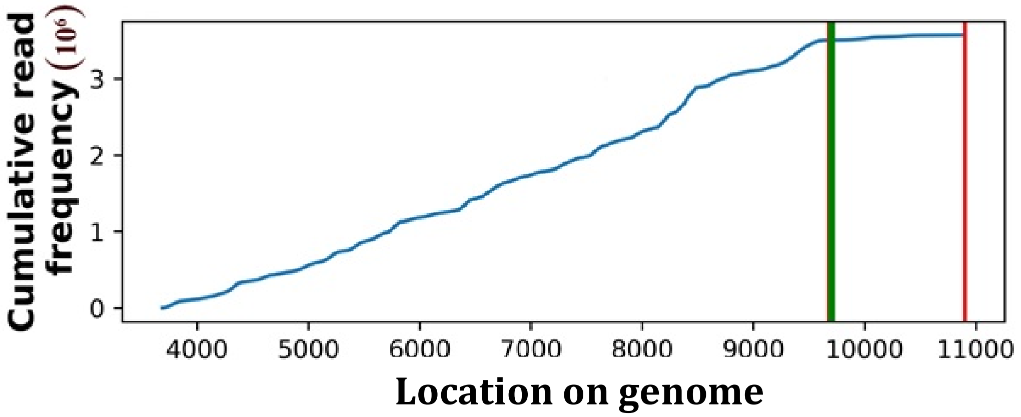

i)

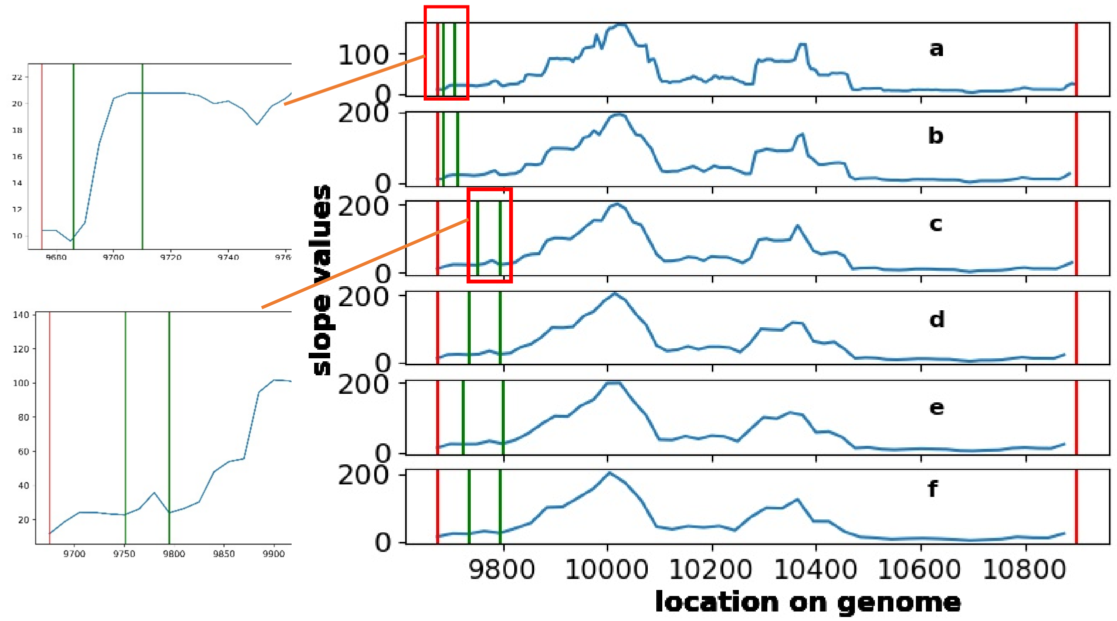

ii)

Fig. S14. Cumulative read frequency distribution plot. Plots show a cumulative read frequency distribution plot for an operon in *S. aureus* in i) and their corresponding slope values in ii) calculated from the cumulative frequency distribution by taking 'w' locations (a-f show w=5,10,15, and so on up to 30) in a window sequentially from the operon shown in a). The x-axis shows the location on the genome and the y-axis shows cumulative read frequency on that location in a) and slope values in b). The red lines mark the start and end of the IR region. The green lines mark the start and end of the first positive slope region. As shown in the inset of plot ii), slope values calculated from smaller size windows like 5 and 10 successfully capture all early inflection points in the curve (eg., points P1 and P2); whereas, increasing sizes further to >15 leads to loss of such points. So, we took window 10 for all further analysis. It is visible from ii) that this window size can detect the early positive slope region closest to the stop codon. If we compare the slope values, the ratio of IR: coding region slopes would range from 0.0001-0.004, showing that even though the slope values are positive in IR, the marginal increase of reads is negligible.

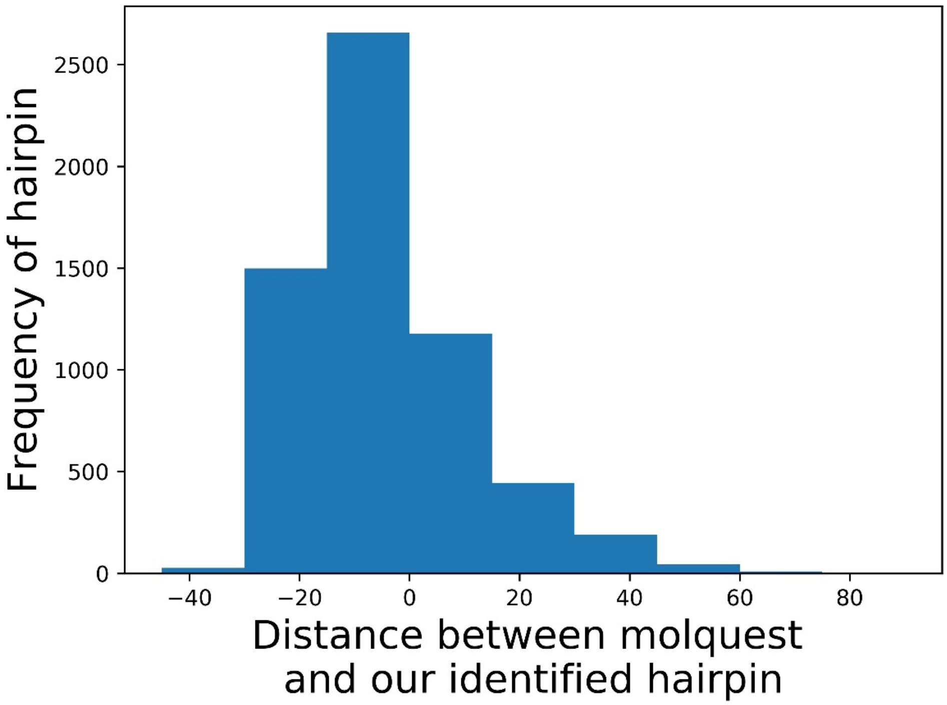

Fig. S15. Comparison with Molquest. The bar diagram illustrates the distance between our *identified* hairpin units and terminator sites reported by Molquest versus the number of cases.

Supplementary File

Supplementary Results

Section R1. Checking for patterns within 25 bases from the stop codon

We took subspecies for different bacteria and found orthologs in them using Orthovenn (Wang et al., 2015; Xu et al., 2019). From there, we took operons where all genes in an operon were conserved as well as present on the same strand. This is shown in Table S8. Now, for all these operons we performed multiple sequence alignment to find regions of conservation, species-wise, using Clustal Omega (Sievers et al., 2011; Sievers & Higgins, 2018). We separately find conservation in coding versus IR region. For this, we find the number of conserved sites in subspecies and divide it by the total coding or IR region, respectively. This analysis is repeated for 0-25 nucleotides from the stop codon as well. As shown in Fig. S10, we see that the coding region has conservation (as expected since these are orthologs), but in complete IR region or for 0-25 nucleotides within stop codon we do not see any conservation, with 76% species having conservation between only 10-50%. This absence of sequence conservation led us to suggest that even with high conservation of coding region, the termination region is distinct across species and subspecies. It is possible that instead of sequence, there is conservation of structure only in these regions. This would require more extensive studies with use of 2D/3D structures for these regions.

References

Sievers, F., & Higgins, D. G. (2018). Clustal Omega for making accurate alignments of many protein sequences. *Protein Science*, *27*(1), 135–145. https://doi.org/10.1002/pro.3290

Sievers, F., Wilm, A., Dineen, D., Gibson, T. J., Karplus, K., Li, W., Lopez, R., McWilliam, H., Remmert, M., Söding, J., Thompson, J. D., & Higgins, D. G. (2011). Fast, scalable generation of high-quality protein multiple sequence alignments using Clustal Omega. *Molecular Systems Biology*, *7*(1). https://doi.org/10.1038/msb.2011.75

Wang, Y., Coleman-Derr, D., Chen, G., & Gu, Y. Q. (2015). OrthoVenn: A web server for genome wide comparison and annotation of orthologous clusters across multiple species. *Nucleic Acids Research*, *43*(W1), W78–W84. https://doi.org/10.1093/nar/gkv487

Xu, L., Dong, Z., Fang, L., Luo, Y., Wei, Z., Guo, H., Zhang, G., Gu, Y. Q., Coleman-Derr, D., Xia, Q., & Wang, Y. (2019). OrthoVenn2: A web server for whole-genome comparison and annotation of orthologous clusters across multiple species. *Nucleic Acids Research*. https://doi.org/10.1093/nar/gkz333
